# MeCP2 mediates transgenerational transmission of chronic pain

**DOI:** 10.1016/j.pneurobio.2020.101790

**Published:** 2020-03-18

**Authors:** Wenjuan Tao, Changmao Chen, Yuping Wang, Wenjie Zhou, Yan Jin, Yu Mao, Haitao Wang, Likui Wang, Wen Xie, Xulai Zhang, Jie Li, Juan Li, Xiangyao Li, Zhen-Quan Tang, Chenghua Zhou, Zhizhong Z. Pan, Zhi Zhang

**Affiliations:** a Department of Physiology, School of Basic Medical Sciences, Anhui Medical University, Hefei 230022, PR China; b Hefei National Laboratory for Physical Sciences at the Microscale, Key Laboratory of Brain Function and Disease of Chinese Academy of Science, Department of Biophysics and Neurobiology, University of Science and Technology of China, Hefei 230027, PR China; c Department of Anesthesiology and Pain Management, The First Affiliated Hospital of Anhui Medical University, Hefei 230022, PR China; d Department of Psychology, Anhui Mental Health Center, Hefei 230026, PR China; e Key Laboratory of Medical Neurobiology of the Ministry of Health of China, Key Laboratory of Neurobiology of Zhejiang Province, Department of Neurobiology, Zhejiang University School of Medicine, Hangzhou 310058, PR China; f Oregon Hearing Research Center, Oregon Health and Science University, Portland, OR 97239, USA; g Department of Anesthesiology and Pain Medicine, The University of Texas MD Anderson Cancer Center, Houston, Texas 77030, USA

**Keywords:** Chronic pain, MeCP2, Primary somatosensory cortex, Glutamatergic neuron, Transgenerational transmission

## Abstract

Pain symptoms can be transmitted across generations, but the mechanisms underlying these outcomes remain poorly understood. Here, we identified an essential role for primary somatosensory cortical (S1) glutamate neuronal DNA methyl-CpG binding protein 2 (MeCP2) in the transgenerational transmission of pain. In a female mouse chronic pain model, the offspring displayed significant pain sensitization. In these mice, MeCP2 expression was increased in S1 glutamate (Glu^S1^) neurons, correlating with increased neuronal activity. Downregulation of Glu^S1^ neuronal MeCP2 in maternal mice with pain abolished offspring pain sensitization, whereas overexpression of MeCP2 in naïve maternal mice induced pain sensitization in offspring. Notably, single-cell sequencing and chromatin immunoprecipitation analysis showed that the expression of a wide range of genes was changed in offspring and maternal Glu^S1^ neurons, some of which were regulated by MeCP2. These results collectively demonstrate the putative importance of MeCP2 as a key regulator in pain transgenerational transmission through actions on Glu^S1^ neuronal maladaptation.

## 1. Introduction

Chronic pain is the leading cause of disability worldwide. The proper treatment for chronic pain, such as neuropathic pain and cancer-induced pain, represents a major challenge in the field. In particular, chronic pain tends to aggregate in families; the offspring of parents with chronic pain are at a high risk of having pain as children, which is indicative of the transgenerational transmission of pain (Higgins et al., 2015; Hoftun et al., 2013; Stone and Wilson, 2016). At present, the mechanisms underlying parent-derived transgenerational transmission of pain remain unclear.

A wide range of heritability has shown that multiple types of clinical pain are strongly heritable, such as migraine pain (≈50 %), lower back and neck pain (35 %–68 %), shoulder and elbow pain (≈50 %), and carpal tunnel syndrome (≈40 %) (Hakim et al., 2003; Smith et al., 2011). Of note, very few types of pain follow the Mendelian transmission model because mutations leading to abnormal pain perception occur in under 1% of the population (Crow et al., 2013; Diatchenko et al., 2007). In fact, a single gene change causing a major impairment in nociception is rare in nature (Kurth et al., 2009; Leipold et al., 2013). Thus, a genetic mutation hypothesis cannot generally explain the transgenerational transmission of pain phenotypes to offspring (Crow et al., 2013; Mogil, 2012). Rather, a summation of small differences in multiple genes is supposed to cause most variations in pain perception. Therefore, the possibility is raised that non-genomic factors, such as epigenetic regulation, might be involved in fine controlling of a population of gene-transcriptional activity in offspring to confer pain phenotypes (Anway et al., 2005; Lane et al., 2014). However, whether and how this occurs in chronic pain transgenerational transmission is entirely unknown.

It has been proposed that a small minority of imprinted genes, which are epigenetically misregulated in parents, can transgenerationally stabilize DNA methylation patterns. These imprinted epigenetic marks, which are passed from parent to progeny on gametes, may shape individual offspring (Daxinger and Whitelaw, 2012; Grossniklaus et al., 2013; Heard and Martienssen, 2014; Lane et al., 2014). The adaptive epigenetic regulation of gene-transcriptional activity has been proposed as molecular mechanisms underlying the etiology of chronic pain development (Bai et al., 2015; Denk and McMahon, 2012; Zhang et al., 2011). Based on the evidence linking the meiotic epigenetic inheritance and transgenerational transmission of behavioral phenotypes, we proposed to address the pathological causes of offspring pain by defining the epigenetic marker-mediated neuronal adaptation under parental chronic pain conditions.

The methyl CpG-binding protein 2 (MeCP2), as an epigenetic marker that is originally known as a transcriptional repressor or activator, contributes to the development and function of the central nervous system (Chahrour et al., 2008; Cohen et al., 2011; Na and Monteggia, 2011). Loss-of-function mutations or duplications of the MeCP2 gene cause Rett syndrome or autism-like behaviors, which can be passed on to the next generation (Amir et al., 1999; Liu et al., 2016; Sztainberg et al., 2015). In particular, abnormal pain sensitivity has been reported in human Rett syndrome patients (Downs et al., 2010), indicating that MeCP2 may be a potential target in investigation of chronic pain transgenerational transmission.

## 2. Material and methods

### 2.1. Animals

In all experiments, C57BL/6 J (purchased from Charles River), *CaMKII*-*Cre* and *Ai14* (RCL-tdT) mice (purchased from Jackson Laboratories) 6–8 weeks old were used. Until the cannula surgery, the mice were housed five per cage in a colony with *ad libitum* access to water and food (standard mouse chow). They were maintained under a 12-h light/dark cycle (lights on from 8:00 a.m. to 8:00 p.m.) at a stable temperature (23 °C–25 °C). All animal protocols were approved by the Animal Care and Use Committee of the University of Science and Technology of China.

### 2.2. Chronic constraint injury (CCI)

The CCI surgery was performed with the mice under anesthesia with isoflurane. The skin and muscle of the left thigh were incised to explore the sciatic nerve: sural, common peroneal, and tibial nerves. After exploration, two loose consecutive ligations were made by 4.0 chromic gut ligatures (Bennett and Xie, 1988). The skin was stitched and dis-infected with iodophor. For the sham surgery, the nerve was isolated but not ligated. Pain thresholds were measured by the paw-withdrawal test on a freely moving animal with the Hargreaves apparatus (IITC Life Science Inc., US) for thermal hyperalgesia, or with von Frey filaments (Stoelting, US) for mechanical allodynia. The pain thresholds were defined by the average of the results of three successive tests. To avoid excessive application of von Frey filaments and of thermal stimuli that elicit aversive behaviors or pain sensitization, the interval between each measurement on the same mouse had to exceed five minutes.

### 2.3. Breeding scheme

All animals bred for transgenerational studies were virgin, experimentally naive C57BL/6J mice. They had been bred in house for at least one or two generations to control for exposures in maternal care, breeding paradigms, early life stress, and shipment differences. To minimize vendor’s effects in experiments, *CaMKII*-*Cre* and *Ai14* mice were not crossed with C57 mice for investigation of transgenerational transmission of pain behavior.

All adult mice (F0), male and female, were randomly divided into the following four groups: female mice with CCI (n = 25); female sham-operated mice (n = 25); male mice with CCI (n = 15); and male sham-operated mice (n = 15). Four weeks after surgery, four group mice were mated with naive mice for 3–7 nights. To minimize male-female interactions (Rodgers et al., 2013), paternal mice were removed from the mating cages when pregnancy was established. At birth, litters were culled to eight pups and litters containing fewer than two pups were excluded from analysis (Mueller and Bale, 2007). There were 171 females (sham offspring n = 82; CCI offspring n = 89) and 163 males (sham offspring n = 78; CCI offspring n = 85) from 50 litters of female-operated mice in the F1 generation. To exclude any specific cage or parental effects, we randomly selected 2–3 offspring mice from each litter in each generation for behavioral tests. To determine whether the maternal chronic pain has significant transgenerational effects on offspring, F1 sham offspring or CCI offspring females were bred with naive males to generate F2 sham offspring (n = 15 litters, n = 56 females and 52 males) or F2 CCI offspring (n = 15 litters, n = 54 females and 53 males). The F3 generation (F3 sham offspring, n = 15 litters, n = 50 females and 51 males; F3 CCI offspring, n = 15 litters, n = 55 females and 52 males) arises from the F2 female offspring crossed with naive mice. The scheme of the breeding paradigm is given in Supplementary Fig. S1A.

### 2.4. Assessment of anxiety-like behavior

#### 2.4.1. Open field test

Mice were placed in one corner of an open field apparatus consisting of a square area (25 × 25 cm^2^) and a marginal area (50 × 50 × 60 cm^3^); the mice were allowed to freely explore their surroundings. The animals’ movement trajectories were recorded for 5 min using EthoVision XT software, which records the number of entries into and the amount of time spent in the central area (Zhou et al., 2019).

#### 2.4.2. Elevated plus maze test

The EPM consists of a central platform (6 × 6 cm^2^), two closed arms (30 × 6 × 20 cm^3^) and two opposing open arms (30 × 6 cm^2^). It was placed 100 cm above the floor. Each mouse was placed in the central platform facing a closed arm and was allowed to explore the maze for five min. The time spent in the open arms and the number of entries into the open arms were analyzed using EthoVision XT software (Noldus) (Zhou et al., 2019).

### 2.5. In vivo two-photon calcium imaging

#### 2.5.1. Cranial window surgery

Mice were anesthetized with sodium pentobarbital (80 mg/kg, i.p.) and immobilized in a stereotaxic apparatus. A circular craniotomy (≈2–3 mm diameter) was made above S1 and then a volume of 250 nl virus (AAV-CaMKIIa-GCaMP6f) was injected into the S1^L2/3^. The craniotomy was covered with 1.2 % agarose, and a round coverglass was cemented to the skull. Custom-designed stainless steel headbars were attached to the skull screw, and dental cement and glue were used to affix both the coverglass and headbar to the skull. The antiphlogistic carprofen (6 mg/kg, i.p.) and enrofloxacin (125 mg/kg, s.c.) were provided before and after surgery, then the mice were taken back to cages until two-photon imaging.

#### 2.5.2. Calcium imaging

Two weeks after implantation, we checked animals that were awake for GCaMP6f fluorescence and Ca^2+^ transient activity. Calcium imaging in awake mobile mice was performed using an upright two-photon microscope (FVMPE-RS, Olympus, Japan) in frame-scan mode. Live images were acquired using a 20 × 0.8 NA macro water objective lens with IR laser (excitation wavelengths of 940 nm). Image acquisition was performed using FV30S-SW (Olympus, Japan) and image datasets were collected at 60 Hz (256 × 256 pixels) in the x–y plane. The typical average power used for imaging S1^L2/3^ GCaMP6f-expressing neurons was 20–30 mW. To determine the calcium signal induced by tail shock stimuli, 2-s current (0.1 mA) stimuli were applied to the base of the tail during the recording of calcium signals (Makino and Komiyama, 2015). Visual stimuli were synchronized and presented to individual image frames using LabState (Anilab Software & Instruments Co., Ltd., China). Tail shock was used on mouse feet but mechanical stimuli were not because non-noxious stimuli on feet cannot reliably induced calcium signal *in vivo*.

#### 2.5.3. Data processing and analysis

Time-series data were imported into ImageJ for further analysis. Imaging data were corrected for mechanical drift using *TurboReg*, and sequential images were used to produce time-lapse movies. The data were analyzed using ImageJ-based ROI analysis. Ring-shaped regions of interest (ROIs) were placed at the body regions of GCaMP6f-expressing neurons. The time-series fluorescence of each cell was measured by averaging all pixels within the ROI, with a correction for neuropil contamination. Calcium transients were considered stimulus-evoked if they occurred no more than 2 s after stimulus delivery and were not associated with any measurable locomotor activity of the animal. Calcium-signal amplitudes were calculated as (F_t_−F_0_)/F_0_ as a function of time, which is the ratio of fluorescent difference (F_t_−F_0_) to basal value (F_0_). The average fluorescence intensity in the baseline period was taken as F_0_ and measured as the average over a 2 s period before the initiation of shock stimulation. Relative change in fluorescence intensity (ΔF/F) normalized to the basal value was calculated after background subtraction. Traces of ΔF/F versus time were generated for each GCaMP6f-expressing neuron. The average ΔF/F values were calculated for statistical significance by repeated measures ANOVA.

### 2.6. Brain slice electrophysiology

#### 2.6.1. Brain slice preparation

All mice were anesthetized with 2% (w/v) sodium pentobarbital (50 mg/kg, i.p) and intracardially perfused with 20 ml oxygenated ice-old *N*-methyl-d-glucamine (NMDG) artificial cerebrospinal fluid (NMDG ACSF) containing (in mM): 1.2 NaH_2_PO_4_, 2.5 KCl, 93 NMDG, 20 HEPES, 25 Glucose, 30 NaHCO_3_, 5 Na-ascorbate, 2 Thiourea, 3 Na-pyruvate, 10 MgSO_4_, 0.5 CaCl_2_, 3 glutathione (osmolarity: 300–305 mOsm/kg, pH: 7.3–7.4). Coronal slices (300 μm) that contained the S1 were sectioned at 0.18 mm/s on a vibrating microtome (VT1200s, Leica, Germany). The brain slices were initially incubated in NMDG ACSF for 10 min at 33 °C, and recovered for at least 1 h at 28 °C in N-2-hydro-xyethylpiperazine-*N*-2-ethanesulfonic acid (HEPES) ACSF containing (in mM): 2.5 KCl, 1.2 NaH_2_PO_4_, 92 NaCl, 30 NaHCO_3_, 25 Glucose, 20 HEPES, 5 Na-ascorbate, 3 Na-pyruvate, 2 Thiourea, 2 MgSO_4_, 2 CaCl_2_, 3 glutathione (osmolarity: 300–305 mOsm/kg, pH: 7.3–7.4). The brain slices were transferred to a slice chamber (Warner Instruments, US) for electrophysiological recording, and were perfused with ACSF that contained (in mM) 2.4 CaCl_2_, 129 NaCl, 3 KCl, 1.3 MgSO_4_, 1.2 KH2PO_4_, 20 NaHCO_3_, and 10 glucose (osmolarity: 300–305 mOsm/kg, pH: 7.3–7.4) at 2.5–3 ml/min. The temperature of the ACSF was maintained at 32 °C by an in-line solution heater (TC-344B, Warner Instruments, US).

#### 2.6.2. Electrophysiological recordings

An infrared (IR)–differential interference contrast (DIC) microscope (BX51WI, Olympus, Japan) equipped with fluorescent fittings was used to visualize neurons in S1 slices. Whole-cell patch-clamp recordings were carried out using a patch-clamp amplifier (MultiClamp 700B Amplifier, Digidata 1440A analog-to-digital converter, US) and pClamp 10.7 software (Axon Instruments/Molecular Devices, US). Patch pipettes consisting of borosilicate glass (VitalSense Scientific Instruments Co., Ltd., Wuhan, China) were pulled to resistances of 5–7 MΩ on a four-stage horizontal puller (P1000, Sutter Instruments, US). The current-evoked action potential firing was recorded in current-clamp mode (I = 0 pA) with internal solution contained (in mM): 130 K-gluconate, 5 KCl, 2 MgCl_2_, 10 HEPES, 0.6 EGTA, 0.3 Na-GTP and 2 Mg-ATP (osmolarity: 285–290 mOsm/kg, pH: 7.2). All recordings were Bessel-filtered at 2.8 kHz and sampled at 100 kHz. Only neurons with series resistance below 30 MΩ and changing < 20 % throughout the recording were used for analysis. All analyses were performed in Clampft version 10.7 (Axon Instruments, US).

### 2.7. Virus injection

Prior to surgery, the mice were fixed in a stereotactic frame (RWD, Shenzhen, China) under a combination of xylazine (10 mg/kg) anesthesia and ketamine (100 mg/kg) analgesia. A heating pad was used to maintain the core body temperature of the animals at 36 °C. A volume of 100–300 nl virus (depending on the expression strength and viral titer) was injected using calibrated glass microelectrodes connected to an infusion pump (RWD, Shenzhen, China) at a rate of 15 nl/min. The coordinates were defined as dorso-ventral (DV) from the brain surface, anterior-posterior (AP) from bregma and medio-lateral (ML) from the midline (in mm).

The AAV-CaMKIIα-MeCP2-2A-mcherry (AAV2/9, 1.12 × 10^12^ vg/ml), AAV-CaMKIIα-mcherry (AAV2/9, 6 × 10^12^ vg/ml), AAV-CaMKIIα-mCherry-mir30-MeCP2 shRNA (AAV 2/9, 2 × 10^13^ vg/ml), and AAV-CaMKIIα-mir30-scramble shRNA-mCherry (AAV2/9, 1 × 10^13^ vg/ml) were purchased from Zhi En Biology (HeFei, China). AAV-CaMKIIα-hChR2 (H134R)-mCherry (AAV2/9, 5.97 × 10^12^ vg/ml) and AAV-CaMKIIα-eNpHR3.0-mCherry (AAV2/9, 8 × 10^12^ vg/ml) were used for optogenetic manipulation three weeks after injection, which were provided by Dr. Fuqiang Xu (Wuhan, China). AAV-CaMKIIa-GCaMP6f (AAV2/9, 5.9 × 10^12^ vg/ml) was purchased from BrainVTA (Wuhan, China). The virus was delivered into the layer 2/3 of the S1 at two sites (first site: AP, −0.40 mm, ML, −1.90 mm; DV, −0.50 mm; second site: AP, −0.50 mm, ML, −1.85 mm, DV, −0.50 mm). Animals with missed injections were excluded.

### 2.8. Optogenetic manipulations

An optical fiber cannula was initially implanted into the S1 of an anesthetized mouse that had been immobilized in a stereotaxic apparatus. The implant was secured to the animal’s skull with dental cement. Chronically implantable fibers (diameter, 200 μm, Newdoon, Hangzhou, China) were connected to a laser generator using optic fiber sleeves. The delivery of blue light (473 nm, 2–5 mW, 10 ms pulses, 20 Hz) or yellow light (594 nm, 5–8 mW, constant) was controlled by a Master-8 pulse stimulator (A.M.P.I., Jerusalem, Israel). The same stimulus protocol was applied in the control group. The location of the fibers was examined after all of the experiments, and data obtained from mice which the fibers outside desired brain region were discarded. Behavioral assays were performed immediately after light stimulation.

In brain slices, optical stimulation was delivered using a laser (Shanghai Fiblaser Technology Co., Ltd., China) through an optical fiber 200 μm in diameter positioned 0.2 mm from the surface of the brain slice. To test the functional characteristics of AAV-CaMKIIα-ChR2, fluorescently labeled neurons that expressed ChR2 were visualized and stimulated with a blue (473 nm, 5–10 mV) laser light using 5-Hz, 10-Hz, or 20-Hz stimulation protocols with a pulse width of 10 ms. The function of eNpH3.0 was assessed by applying sustained yellow (594 nm, 1–5 mV, 200 ms) laser light stimulation.

### 2.9. Immunofluorescence staining

The mice were anesthetized with sodium pentobarbital (50 mg/kg, i.p.) and sequentially perfused with saline and 4% (w/v) paraformaldehyde (PFA). The brains were subsequently removed and post-fixed in 4% PFA at 4 °C overnight. After cryoprotection of the brains with 30 % (w/v) sucrose, coronal sections (40 μm) were cut on a cryostat (Leica CM1860, Germany) and used for immunofluorescence. The sections were firstly treated with citrate antigen retrieval solution, and then incubated in 0.3 % (v/v) Triton X-100 for 0.5 h, blocked with 5% donkey serum for 1 h at room temperature, and incubated with primary antibodies, including anti-MeCP2 (1:500, rabbit, Cell Signaling) and anti-vGluT2 (1:50, mouse, Millipore) at 4 °C for 24 h, followed by the corresponding fluorophore-conjugated secondary antibodies, including Alexa Fluor 488 donkey anti-rabbit IgG (1:500, Invitrogen), Alexa Fluor 594 donkey anti-mouse IgG (1:500, Invitrogen), and Alexa Fluor 594 donkey anti-rabbit IgG (1:500, Invitrogen) for 2 h at room temperature. Immunofluorescence staining for MeCP2, vGluT2 and their overlap from randomly selected sections (n = 3–4 sections from each mice). Fluorescence signals were visualized using a Zeiss LSM710 microscope, and further analyzed using TissueQuest software (TissueGnostics, US).

### 2.10. Fluorescence-activated cell sorting (FACS)

#### 2.10.1. Cell dissociation

Mice were anesthetized with 2% (w/v, i.p.) sodium pentobarbital and then perfused with 20 ml oxygenated ice-old NMDG ACSF. Brains were dissected and sectioned in the coronal plane at 400 μm on a vibratome (VT1200s, Leica, Germany) in a chilled NMDG ACSF solution, and bubbled with 5% CO_2_/95 % O_2._ The S1^L2/3^ tissue was extracted and dissociated using the Papain Dissociation System (Worthington Biochem, US). The digested tissue was triturated with three Pasteur pipets of decreasing tip diameter. To remove excess debris, the cell suspension was subjected to centrifugation on an AOI discontinuous gradient (per Papain Dissociation System protocol) at 900 rpm for 8 min at room temperature. Cell pellets were re-suspended in FACS buffer (L15−CO2 without phenol, 1 × Pen-Strep, 10 mM Hepes, 25 μg/ml DNase, 1 mg/ml BSA) and filtered through a 70 μm metal mesh.

#### 2.10.2. FACS purification of Glu^S1L2/3^ neurons

The dissociated cells were cross-linked with 37 % formaldehyde (1% final concentration) at room temperature for 10 min and then re-suspended in 1 ml of PBS for labeling. Tween-20 was added to the sample (0.05 % final concentration), and gently vortexed. After washing at 2000 rpm for 5 min, cells were incubated with primary antibody against Glutamate (1:200, Sigma) and 1% bovine serum albumin (BSA) for 1 h at 4 °C. Cells were then washed and incubated with the second antibody of Alexa Fluor488 (1:300, Invitrogen) for 30 min at 4 °C. Cell pellets were diluted in the PBS buffer and glutamate-positive cells were isolated on the BD FACS Aria II Cell Sorter. The following controls were employed to ensure the optimal criteria for sorting: unstained cells, and cells in which the primary anti-glutamate antibody was omitted to control for potential background arising from the Alexa Fluor488 s antibody. Glutamate cells (≈50,000) were collected into PBS for chromatin or RNA extraction. For mice with S1 infusion of AAV-CaMKIIα-MeCP2-mCherry, the mCherry labeled neurons were directly used for sorting without staining, which were used to perform Western blotting.

### 2.11. Western blot

Glutamate cells collected from FACS were lysed in RIPA buffer (50 mM Tris−HCl, 150 mM NaCl, 0.1 % SDS, 1% Triton X-100, 0.5 % sodium deoxycholate, and protease inhibitor cocktail) for 30 min. Following centrifugation at 12,000 g at 4 °C for 10 min, the supernatant was used to measure protein concentration by Pierce BCA Protein Assay Kit (Thermo). Fifteen μg of protein was mixed with SDS sample buffer and were boiled for 10 min. Then, protein samples were separated on 5% stacking gel with 70 V for 20 min and 8% separating gel with 110 V for 1 h. Proteins were transferred with NC membranes (Millipore) for 50 min at a constant current of 280 mA. After immersion in blocking solution for 1 h at room temperature, the membranes were incubated with the primary antibodies MeCP2 (1:1000, Cell Signaling) and GAPDH (1:3000, Cell Signaling, 5174S) overnight at 4 °C. Membranes were then incubated in peroxidase-labeled goat anti-rabbit secondary antibody (1:5000, Thermo Scientific) at room temperature for 2 h. The protein bands were detected with Pierce^™^ ECL Plus Substrate (Thermo Scientific) and analyzed with ImageJ software.

### 2.12. Single-cell RNA sequencing

#### 2.12.1. Cell harvesting

The brain slices containing S1 were prepared according to the above method for electrophysiological recording. The tdTomato-expressing glutamatergic (CaMKII-tdTOM) neurons were randomly selected and aspirated into a glass electrode for single-cell RNA extraction as described previously (Fuzik et al., 2016; Li et al., 2016). Briefly, the entire soma of each recorded neuron was aspirated into the micropipette slowly (≈3–5 min) by applying mild negative pressure. To minimize the changes in gene expression in different neurons, this procedure was finished within 2 h under clean-room conditions.

#### 2.12.2. RNA isolation and library construction

A single Glu^S1L2/3^ neuron was gently transferred into lysis buffer, and a SMART-Seq^™^ v4 Ultra^™^ Low Input RNA Kit (Clontech) was directly used to reverse transcription and cDNA amplification in the cell lysate. Then, the sample (50 μl) was subjected to cDNA purification according to the manufacturer’s protocol. The RNA integrity was checked by Agilent Bioanalyzer 2100 (Agilent technologies). The purified cDNA was sheared by Covaris S2 into 150–350 bp fragments. The sequencing library was constructed by over 10 ng of the purified cDNA from each neuron with Ovation Ultralow Library System V2 (Nugen) following the manufacturer’s protocol.

#### 2.12.3. Sequence alignment and data analysis

The cDNA library was paired-end sequenced using the Illumina sequencing platform (HiSeq 2500). The library was sequenced to a ≈150 bp length. The short sequences (length < 25 bp), low-quality bases (quality < 20), and adaptor sequences were removed to filter the raw reads using Seqtk. To improve the utilization of reads, the RNA-seq reads were mapped to the mouse mm10 genome with two gaps, two mismatches and one multihit by Hisat2. The gene expression was quantified by the StringTie v1.3.0 after genome mapping. The value of gene expression was normalized by FPKM with a geometric algorithm adjustment. We used edgeR to identify differentially expressed genes in all the transcriptomic data (Robinson et al., 2010). Gene ontology (GO)–enrichment analysis (clusterProfiler R package) was performed as described previously (Yu et al., 2012).

### 2.13. Quantitative real-time PCR

To verify single-cell RNA-seq data, FACS-sorted Glu^S1L2/3^ neurons as described above were used to perform real-time PCR. Total RNA was extracted from Glu^S1L2/3^ neurons with Trizol reagent (Sangon Biotech) and reverse transcription was performed using a GoScript^™^ Reverse Transcription kit (Promega, A5001) according to the manufacturer’s protocol. Quantitative real-time PCR reactions were performed using 1–10 ng of cDNA templates on an ABI Stepone system (Applied Biosystems). GAPDH mRNA quantification was used as a control for normalization. Fold differences of mRNA levels over controls were calculated by the 2^−ΔΔCt^ method. Each PCR reaction was repeated at least twice independently. Sequences of the primers (Sangon Biotech) used in PCR are provided in Supplementary Table 9.

### 2.14. Chromatin immunoprecipitation (ChIP)

The fixed cells, as described in FACS, were lysed and pooled once more to obtain sufficient material for downstream analysis. The extracted chromatin was sheared in a Bioruptor Plus UCD-300 (Belgium) with eight repeats of 5-min cycles (30 s on, 30 s off) to obtain DNA fragments of 100–500 bp, and ten percent of the lysate was used as the “input” control for normalization later. The lysate was then diluted 5-fold in ChIP dilution buffer (50 mM Tris−HCl pH 8.0, 150 mM NaCl, 2 mM EDTA, 1% TritonX-100, 0.01 % SDS, and proteinase inhibitor cocktail), and precleared with Protein A/G Dynabeads (Invitrogen) for 2 h under constant rotation at 4 °C. The MeCP2 antibody and non-immunized IgG were added to ChIP reactions, then incubated at 4 °C under constant rotation for 16 h. The next day, 20 μl of Protein A/G Dynabeads was added, and the sample was rotated for 1.5 h at 4 °C. Beads were further washed sequentially with ice-cold buffers by adding proteinase inhibitors at room temperature. After removal of the incubated antibody solution, the beads and input sample were re-suspended with 200 μl ChIP elution buffer (10 mM Tris−HCl pH 8.0, 110 mM NaCl, 1 mM EDTA, 1% SDS, 40 μg Proteinase K), and incubated at 42 °C for 3 h, and reversed cross-linked at 65 °C overnight on a rotator. Chip DNA and input DNA was recovered by phenol/chloroform extraction and ethanol precipitation, and the concentration and quality was detected by Qubit^®^ 2.0 Fluorometer and Agilent 2100 (Agilent technologies).

The amount of MeCP2 binding to gene promoters and gene bodies was measured by quantitative real-time PCR. Sequences of the primers (Sangon Biotech) used in PCR are provided in Supplementary Table 5. Amplifications were run in triplicate with SYBR^®^ Green PCR Master Mix kit (Vazyme), and each reaction was repeated at least twice independently. The data were analyzed as described previously (Zhang et al., 2011).

### 2.15. Functional magnetic resonance imaging (fMRI)

#### 2.15.1. Subjects

Fifty-four female right-handed participants whose parents suffered from chronic pain were recruited in this study. All participants were of the Han Chinese ethnicity. Written informed consent was obtained from all the participants and data—including parental and offspring generations—were collected *via* pre-imaging questionnaire packets that included the following: (1) a general demographic and medical history (including pain duration) survey, (2) the Hamilton depression rating scale (HAMD) (Hamilton, 1960; Williams, 1988), and (3) the McGill pain questionnaire (Melzack, 1975). Inclusion criteria for individuals were as follows: (1) people aged 18 and 70, and (2) chronic lower back pain diagnosed by Chinese versions of the Nordic Musculoskeletal and Roland-Morris Questionnaires and by physical examinations (Takekawa et al., 2015; Yi et al., 2012). Exclusion criteria for individuals were as follows: (1) chronic pain of parental generation attributable to the after-effects of cancer, prosthesis, surgery, and radiotherapy, (2) metal implants such as cardiac pacemaker, heart stent, artificial teeth, and hearing aid, (3) presence of claustrophobia, and (4) psychiatric and cognitive disorders, including brain injury and epilepsy history. All patients in this study were recruited from the First Affiliated Hospital of Anhui Medical University (Hefei, China), which was diagnosed according to the McGill Pain Questionnaire (Melzack R). Our research complied with the Code of Ethics of the World Medical Association (Declaration of Helsinki) on human subjects. This study was approved by the ethics committee of the First Affiliated Hospital of Anhui Medical University.

#### 2.15.2. MRI scanning procedures

Each participant received a resting-state scan in a 3.0-T scanner (GE Signa HDx, General Electric, US) with the head fixed by foam pads to minimize head motion. T1-weighted structural images were acquired with a three-dimensional spoiled-gradient recalled-acquisition sequence (3D-SPGR, Time of Repetition, 7.876 ms; Time of Echo, 3.06 ms; slice thickness, 1.2 mm; field of view, 22 cm × 22 cm; resolution, 1 × 1 × 1 mm^3^; Time of inversion, 400 ms). T2*-weighted functional imaging was performed using an echo planar imaging sequence (EPI, Time of Repetition, 2000 ms; Time of Echo, 22.5 ms; slice thickness, 4.0 mm; field of view, 22 cm × 22 cm; skip between slices, 0.6 mm; matrix, 64 × 64; flip angle, 30 °C; voxel size, 3.75 × 3.75 × 4 mm^3^). For each participant, 33 axial slices without gaps were obtained in an interleaved-ascending order covering the entire brain, and resting-state fMRI data (240 volumes, 8 min) was collected.

#### 2.15.3. Image preprocessing

The fMRI data were pre-processed using Analysis of Functional NeuroImages (AFNI, Medical College of Wisconsin, WI, US). We performed motion correction, Talairach registration, intensity correction, brain extraction, and normalization based on a Gaussian Classifier Atlas. The data with head motion over 2 mm or 2° were excluded. For head motion, no significant difference was observed between groups, nor was any significant difference observed after regressing out head motion. We defined the white matter signal, cerebrospinal fluid signal, global signal, and 24 Friston-motion parameters as nuisance regressors to control for non-neuronal activation. Surface-based smoothing with a full-width at half-maximum (FWHM) of 6 mm Gaussian kernel was applied to remove high-frequency noises. All normalized functional images were resampled by 3.0 × 3.0 × 3.0 mm^3^ voxels.

#### 2.15.4. Amplitude of low-frequency fluctuation (ALFF) analysis

ALFF analysis was performed using Resting-State fMRI Data Analysis Toolkit (REST, http://restfmri.net/forum/index.php) tool-boxes under MATLAB 2014a (Mathworks, US). The REST toolbox calculated the square root of the power spectrum of blood oxygenation level dependent (BOLD) signals from each voxel and computed the sum of frequencies in the low frequency band (0.01–0.08 Hz) (Yu-Feng et al., 2007). A two sample *t*-test was used to analyze the differences of ALFF between groups.

### 2.16. Quantification and statistical analysis

We conducted simple statistical comparisons using Student’s *t* test. ANOVA (one-way and two-way) and *post hoc* analyses were used to statistically analyze the data from the experimental groups with multiple comparisons. All data are here expressed as the mean ± SEM, and significance levels are indicated as *P < 0.05, **P < 0.01, and ***P < 0.001. OriginPro 2017 software (Origin Lab Corporation, US) and GraphPad Prism 5 (Graph Pad Software, Inc., US) were used for the statistical analyses and graphing. Offline analysis of the data obtained from electrophysiological recordings was conducted using Clampfit software version 10.7 (Axon Instruments, Inc., US).

## 3. Results

### 3.1. The transgenerational transmission of pain sensitization

**Fig. 1.**
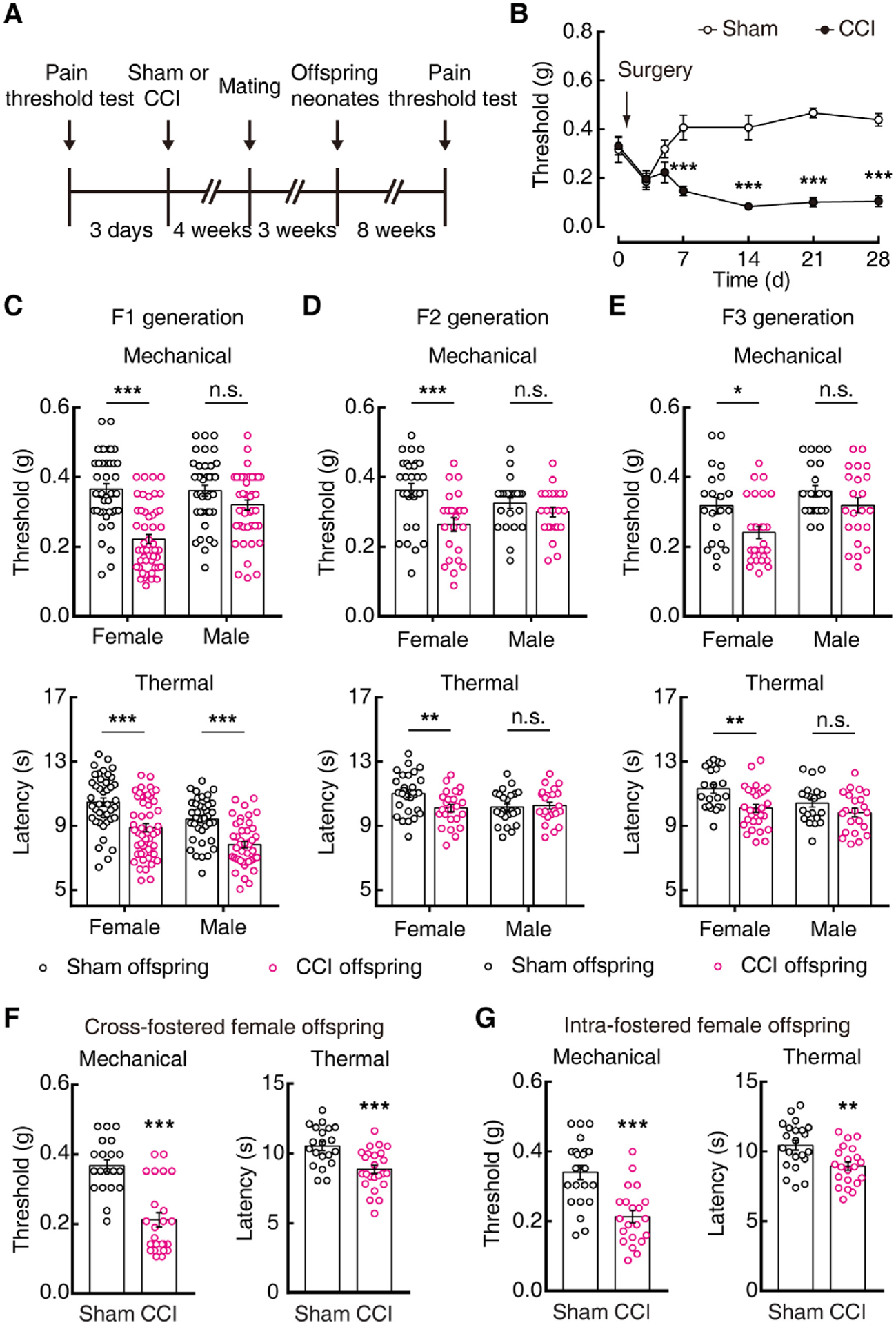
Maternal-persistent pain elicits pain hypersensitivity in female offspring. (A) Experimental timeline of mouse-breeding scheme. (B) Time course of CCI-induced sensory pain in female mice. (C–E) The threshold of mechanical (up) and thermal (down) pain in female and male mice of the first filial generation (F1) (C: sham offspring, n = 41 females and 38 males; CCI offspring, n = 50 females and 44 males. two-way ANOVA, mechanical, interaction *F*_(1, 169)_ = 11.89, *P* < 0.001; thermal, interaction *F*_(1, 169)_ = 0.0002, *P* = 0.988), second filial generation (F2) (D: sham offspring, n = 28 females and 23 males; CCI offspring, n = 24 females and 23 males. two-way ANOVA, mechanical, interaction *F*_(1, 94)_ = 4.249, *P* = 0.042; thermal, interaction F *F*_(1, 94)_ = 4.949, *P* = 0.029) and third filial generation (E: sham offspring, n = 21 females and 21 males; CCI offspring, n = 28 females and 22 males. two-way ANOVA, mechanical, interaction *F*_(1, 88)_ = 0.843, *P* = 0.361; thermal, interaction *F*_(1, 88)_ = 1.307, *P* = 0.256) from sham and CCI maternal mice. (F) Pain threshold of cross-fed F1 female offspring from sham and CCI mice. (G) Pain threshold of F1 female mice internally bred by foster mothers. Data are presented as mean ± SEM. For statistical analyses, see Supplementary Table 9. n.s., not significant; *P < 0.05, **P < 0.01, ***P < 0.001.

To identify the molecular mechanism underlying the transgenerational transmission of pain, it is necessary to consider the behavior in animals that resembles pain sensitization from parents. We employed a well-established chronic constriction injury (CCI) of the sciatic nerve-induced neuropathic pain model in mice (Fig. 1A, B). Interestingly, pain sensitization was observed in female offspring from CCI maternal mice (Fig. 1C), but not from paternal mice (Supplementary Fig. 1B–D). These phenotypes were observed in second and third generation offspring as well (Fig. 1D, E). To rule out the possibility that the offspring pain sensitization is from the empathy of maternal pain (Langford et al., 2006), the CCI and sham maternal mice were exchanged to feed their neonates, and normal-bred foster mothers were used to feed pups to maintain consistency between maternal care. A similar pain sensitization phenotype in CCI female offspring was observed (Fig. 1F, G). The male offspring only displayed slight decreases in thermal responsiveness in F1 generation, but not mechanical pain thresholds (Fig. 1C). Previous studies have reported that neuropathic pain can induce anxiety-like behavior in mice (Jin et al., 2019). We found that CCI female offspring also displayed readily visible anxiety-like behavior (Supplementary Fig. 2A, B). These results suggest that maternal chronic pain has significant transgenerational effects on female offspring, which is similar to what occurs in humans (Hoftun et al., 2013; Kaasboll et al., 2012). Thus, the female offspring from maternal mice were further investigated in the present study.

### 3.2. Increased S1 activity in patients with chronic pain

To identify the brain regions that may be involved in pain transgenerational transmission, we recruited a cohort of 54 female offspring volunteers originating from 54 parental generations with chronic lower back pain, which is one of the most prevalent types of chronic pain (Stubbs et al., 2016). Among the 54 offspring, 35 volunteers were diagnosed with chronic pain. Using resting-state fMRI, we found that the activity in several brain areas was increased in the 35 pain offspring when compared with that of 19 offspring without pain (Supplementary Fig. 3A and Supplementary Table. 1). In particular, the primary somatosensory cortex (S1) was dramatically activated (Supplementary Fig. 3B). As the S1 is the terminal site to integrate pain-related information for sensory-discriminative dimensionality of pain (Liu et al., 2018; Vierck et al., 2013; Zhuo, 2008), we next focused on the role of S1 adaptation in the transgenerational transmission of pain.

### 3.3. Sufficient role of increased Glu^S1L2/3^ neuronal activity for offspring pain sensitization

**Fig. 2.**
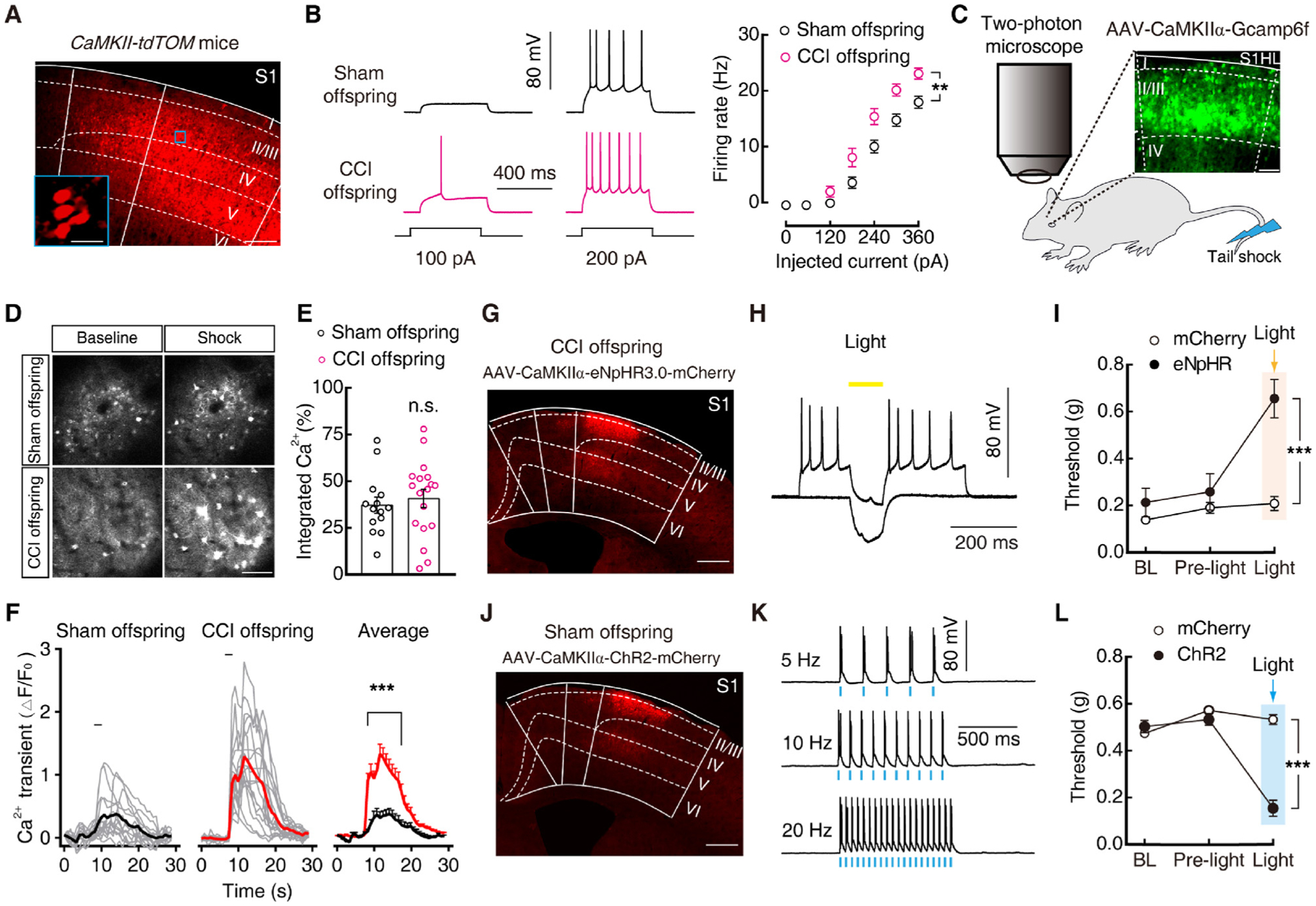
Increased Glu^S1L2/3^ neuronal activity is required for pain hypersensitivity in CCI offspring. (A) Confocal images of Glu^S1L2/3^ neurons from a CaMKII-tdTOM mouse. The blue box depicts the area shown in the boxes of the S1^L2/3^. Scale bars: 100 μm (right) and 20 μm (left). (B) Sample traces (left) and statistical data (right) for action potential firings recorded from S1^L2/3^ tdTOM-expressing neurons in sham and CCI offspring. (C) Schematic paradigm of the *in vivo* two-photon calcium imaging, and a representative image of the S1^L2/3^ two weeks after AAV-CaMKIIα-GCaMP6f injection. Scale bar: 50 μm. (D) Representative *in vivo* S1 calcium images showing AAV-CaMKIIα-GCaMP6f-expressing neurons during rest and evoked by 2 s tail shock in sham and CCI offspring. Scale bar: 50 μm. (E) Average total integrated Ca^2+^ activity of Glu^S1L2/3^ neurons over 10 s before tail shock stimuli. (F) Time course of the amplitude of the calcium transients evoked by shock (0.1 mA) applied to the base of the tail from sham offspring and CCI offspring mice. Each trace is a response from a single GCaMP6f-expressing neuron. All data for calcium transients are expressed as the fold of baseline calcium transient (ΔF/F_0_). Black bars indicate when stimuli were applied. (G) Illustration of viral injection of AAV-CaMKIIα-eNpHR3.0-mCherry in the S1^L2/3^ of CCI offspring. Scale bar, 500 μm. (H) Representative traces of currents from AAV-CaMKIIα-eNpHR3.0-mCherry-expressing neurons evoked by photostimulation (594 nm) in the S1 slice. (I) Effects of photostimulation (594 nm) in the S1^L2/3^ on pain threshold in CCI offspring with S1^L2/3^ infusion of AAV-CaMKIIα-mCherry (mCherry) or AAV-CaMKIIα-eNpHR3.0-mCherry (eNpHR). (J) Representative images of viral expression of AAV-CaMKIIα-ChR2-mCherry in the S1^L2/3^ of sham offspring. Scale bar, 500 μm. (K) Sample traces of action potentials evoked by 473 nm light (blue bars) recorded from S1^L2/3^ AAV-CaMKIIα-eNpHR3.0-mCherry-expressing neurons in the S1 slice. (L) Behavioral effects of photostimulation (473 nm) in the S1^L2/3^ of mice with S1^L2/3^ infusion of AAV-CaMKIIα-mCherry (mCherry) or AAV-CaMKIIα-ChR2-mCherry (ChR2) on pain threshold. Data are presented as mean ± SEM. For statistical analyses, see Supplementary Table 9. n.s., not significant; **P < 0.01, ***P < 0.001.

The layers 2 and 3 of S1 (S1^L2/3^), which consists of approximately 80 % glutamatergic neurons (Markram et al., 2004), forms the super-ficial cortical layer and distributes outputs to other areas (Liu et al., 2018; Navratilova and Porreca, 2014; Zhuo, 2008). Previous studies have reported that c-Fos-labeled neurons became densely increased in layers 2 and 3 and less increased in deep layers 4 and 5 of the S1 in a persistent pain model (Chang et al., 2008), and the spontaneous activity of Glu^S1L2/3^ neurons increased under chronic pain conditions (Eto et al., 2011). For these reasons, we chose layers 2 and 3 of the S1 for experiments. We determined the S1^L2/3^ glutamatergic (Glu^S1L2/3^) neuronal activity by whole-cell recordings in brain slices. To visualize glutamatergic neurons, *Ca*^*2+*^*/calmodulin-dependent protein kinase II alpha* (*CaMKIIα*, an enzyme in glutamatergic neurons)-*Cre* mice were crossed with *Ai14* (RCL-tdT) mice to produce transgenic mice with red tdTomato-expressing glutamate (CaMKII-tdTOM) neurons (Fig. 2A). Using whole-cell recordings in brain slices, we found that both CCI maternal mice and their female offspring had more spikes than control mice (Fig. 2B and Supplementary Fig. 4A, B), but male offspring did not (Supplementary Fig. 5A, B). The difference was not observed in glutamatergic neurons from layer 5 (Supplementary Fig. 6A, B). To monitor calcium activity in conscious mice, we injected AAV expressing a genetically encoded calcium indicator, GcaMP6f, which is driven by *CaMKIIα* promoter (AAV-CaMKIIα-GCaMP6f), into the S1^L2/3^ (Fig. 2C). *In vivo* two-photon calcium imaging showed that the stimuli-evoked average Ca^2+^ transients in activated S1^L2/3^ neurons from CCI offspring were significantly higher than in those from sham offspring (Fig. 2D, F and Supplementary Movie 1–2). No difference was observed in baseline of Ca^2+^ activity before stimulation in the two experimental groups (Fig. 2E).

Given the increased Glu^S1^ neuronal activity in CCI offspring, we subsequently aimed to investigate whether inhibition of the Glu^S1L2/3^ neuronal activity restored pain sensitization. We infused AAV expressing eNpHR3.0-mCherry driven by a neuronal *CaMKIIα* promoter (AAV-CaMKIIα-eNpHR3.0-mcherry) into the S1^L2/3^ to selectively suppress the activity of Glu^S1L2/3^ neurons (Fig. 2G, H). Optical inhibition of Glu^S1L2/3^ neurons significantly reduced pain sensitization of the CCI offspring (Fig. 2I). In addition, in naïve mice, we injected AAV expressing channelrhodopsin-2 (ChR2) driven by the *CaMKIIα* promoter (AAV-CaMKIIα-hChR2 (H134R)-mCherry) into the S1^L2/3^ to selectively activate Glu^S1L2/3^ neurons (Fig. 2J, K). Optical stimulation in the S1^L2/3^ produced pain sensitization in these naïve mice (Fig. 2L). These results suggest that increased Glu^S1L2/3^ neuronal activity is sufficient to prime pain sensitization in offspring from chronic pain mice.

### 3.4. Glu^S1L2/3^ neuronal MeCP2 mediates pain sensitization in offspring

**Fig. 3.**
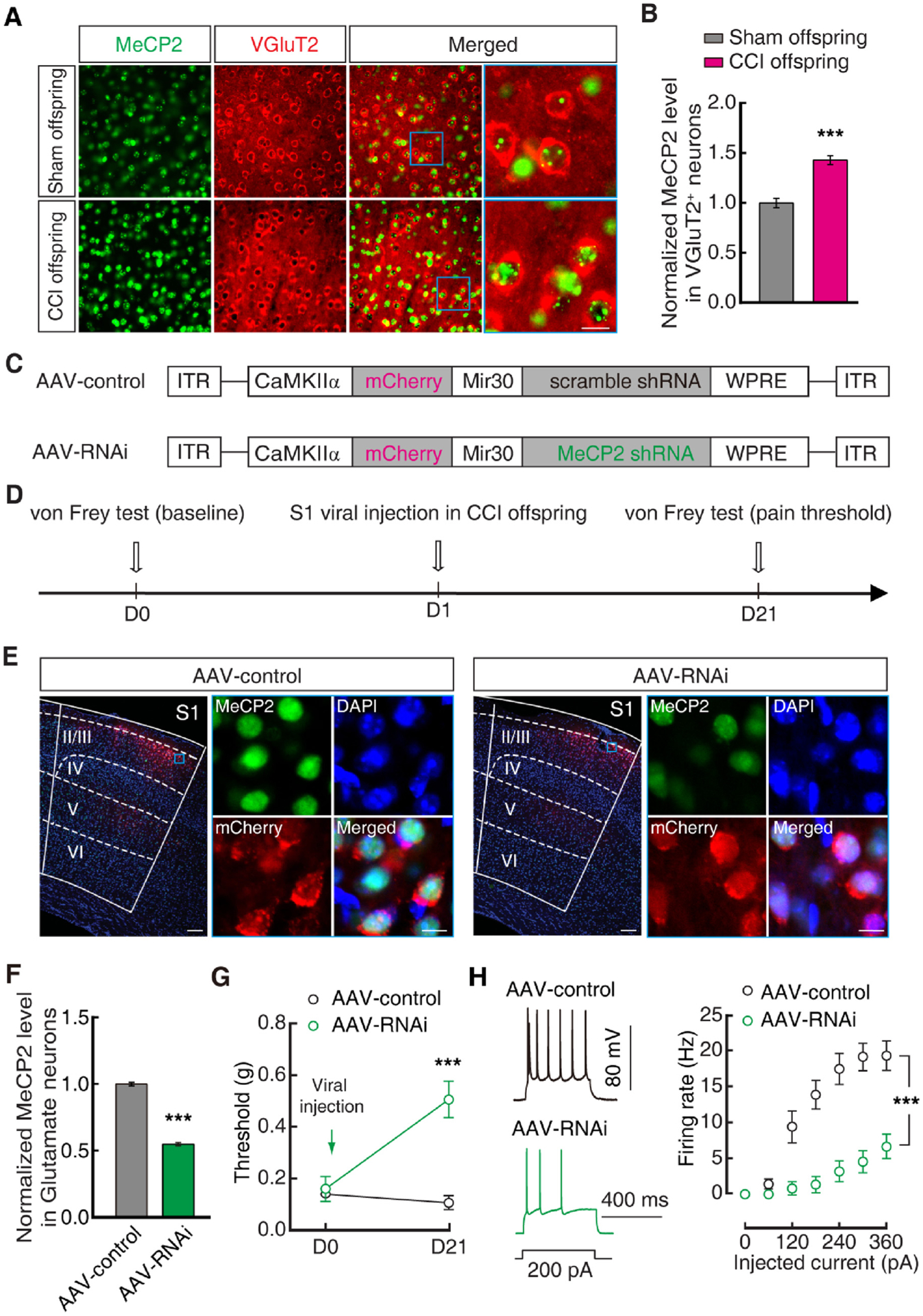
Knockdown of Glu^S1L2/3^ neuronal MeCP2 reduces pain hypersensitivity in offspring. (A) Fluorescent images showing S1^L2/3^ sections from sham and CCI offspring stained for MeCP2 (green) and VGluT2 (red). Scale bar: 10 μm. (B) Statistical data of MeCP2 levels in Glu^S1L2/3^ normalized to sham control. (C) Schematics of AAV vectors engineered to express a control construct (top) or RNAi (bottom) form of MeCP2. ITR, inverted terminal repeats; CaMKIIα, *α-calcium*/*calmodulin-dependent protein kinase II* promoter; Mir30, microRNA; WPRE, woodchuck hepatitis virus posttranscriptional regulatory element. (D) Experimental paradigm for behavioral testing of CCI offspring after S1^L2/3^ viral injection. (E and F) Fluorescent images (E) and statistical data (F) of MeCP2 levels in Glu^S1L2/3^ neurons three weeks after S1^L2/3^ infusion of AAV-control or AAV-RNAi injection in CCI offspring. Data were normalized to AAV-control. Scale bars: 100 μm (left), 10 μm (right). The blue boxes depict the area shown in the boxes of the S1^L2/3^. (G and H) Effects of S1^L2/3^ infusion of AAV-control or AAV-RNAi in CCI offspring on pain behavior (G) and Glu^S1L2/3^ neuronal action potential firings (H). Data are presented as mean ± SEM. For statistical analyses, see Supplementary Table 9. ***P < 0.001.

**Fig. 4.**
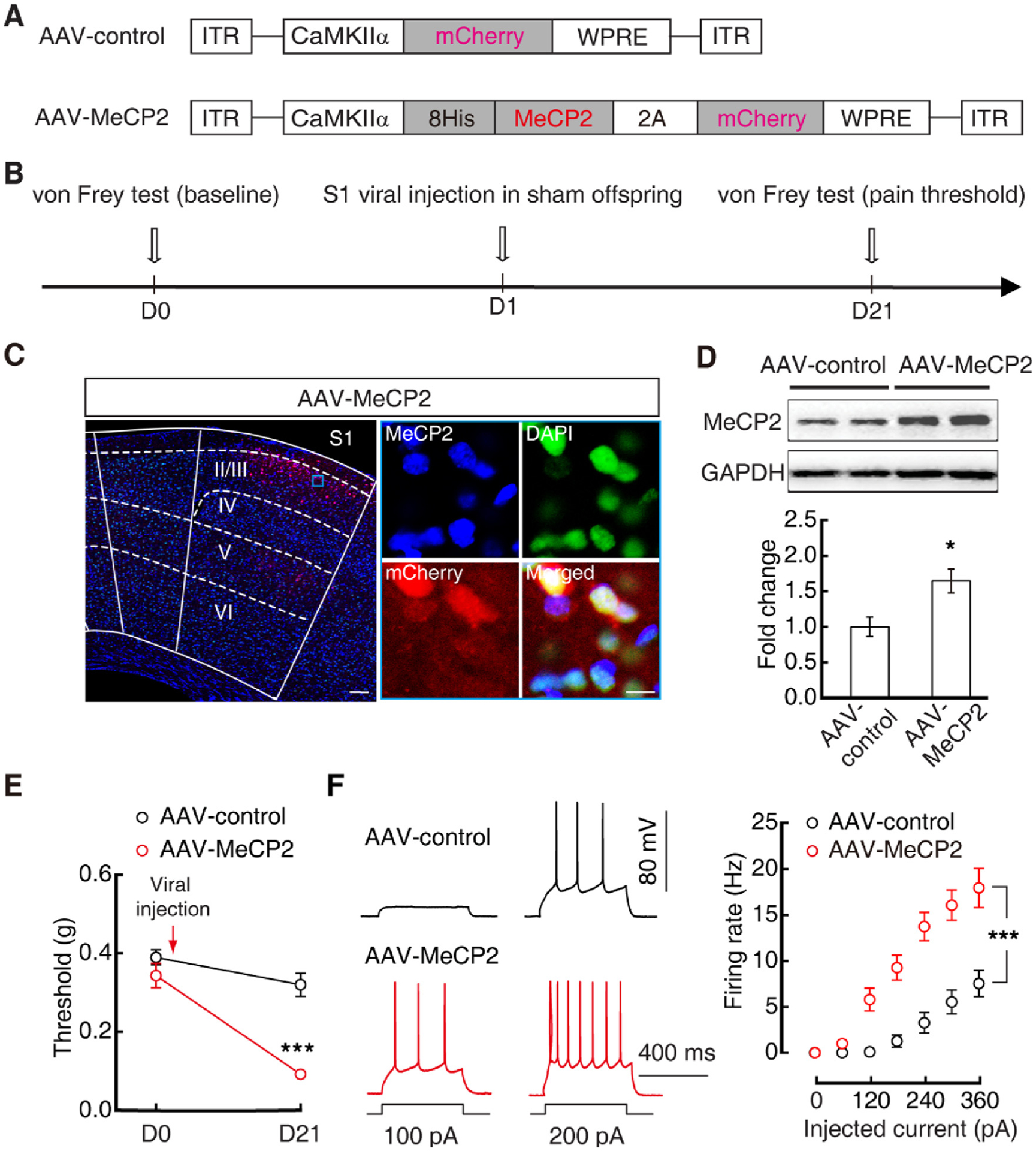
Overexpression of MeCP2 in Glu^S1L2/3^ neurons increases pain hypersensitivity in sham mice. (A) Schematics of AAV vectors engineered to overexpress a control construct or MeCP2. 2A: viral 2A linker peptide allowing translation of multiple unfused proteins. (B) Experimental paradigm for behavioral test and viral injection on sham offspring. (C) Fluorescent images of S1^L2/3^ AAV-MeCP2 expression. Scale bars: 100 μm (left), 10 μm (right). (D) Western blots (top) and statistical data (bottom) of MeCP2 protein levels in S1^L2/3^ neurons sorted by FACS from sham offspring three weeks after S1^L2/3^ injection of AAV-control or AAV-MeCP2. Protein levels were normalized to AAV-control mice. (E and F) Effects of S1^L2/3^ injection of AAV-control or AAV-MeCP2 in sham offspring on pain behavior (E) and Glu^S1L2/3^ neuronal action potential firings (F). Data are presented as mean ± SEM. For statistical analyses, see Supplementary Table 9. *P < 0.05, ***P < 0.001.

Previous research has shown that MeCP2 finely controls substantial gene expression and functions in synaptic plasticity and abnormal pain behavior (Hou et al., 2015; Krishnan et al., 2017; Zhang et al., 2014), dysfunction of which probably generates germline transmission (Liu et al., 2016). We found that the protein level of MeCP2 was significantly higher in Glu^S1L2/3^ neurons in both CCI maternal mice (Supplementary Fig. 4C, D) and their female offspring (Fig. 3A, B), but not in male offspring (Supplementary Fig. 5C, D) when compared with that of sham control mice. To investigate the function of MeCP2, we used *CaMKIIα* promoter-driven RNA interference (RNAi) viral vectors (AAV2/9) to knockdown the Glu^S1L2/3^ neuronal MeCP2 protein level (Fig. 3C). Three weeks after S1^L2/3^ injection of MeCP2 RNAi virus (AAV-RNAi) in CCI offspring (Fig. 3D), Glu^S1L2/3^ neuronal MeCP2 levels were reduced to ≈54 % of those in the AAV control group (AAV-control) (Fig. 3E, F); the pain threshold was increased (Fig. 3G), accompanied by decreased Glu^S1L2/3^ neuronal activity (Fig. 3H). To further study the role of MeCP2 in offspring pain sensitization, we used viral vectors (AAV2/9) driven by the *CaMKIIα* promoter to overexpress MeCP2 in Glu^S1L2/3^ neurons (Fig. 4A, B). Three weeks after S1^L2/3^ infusion of MeCP2-expressing virus (AAV-MeCP2) in sham offspring (Fig. 4C, D), mice displayed significant pain sensitization (Fig. 4E), accompanied by increased Glu^S1L2/3^ neuronal activity (Fig. 4F).

**Fig. 5.**
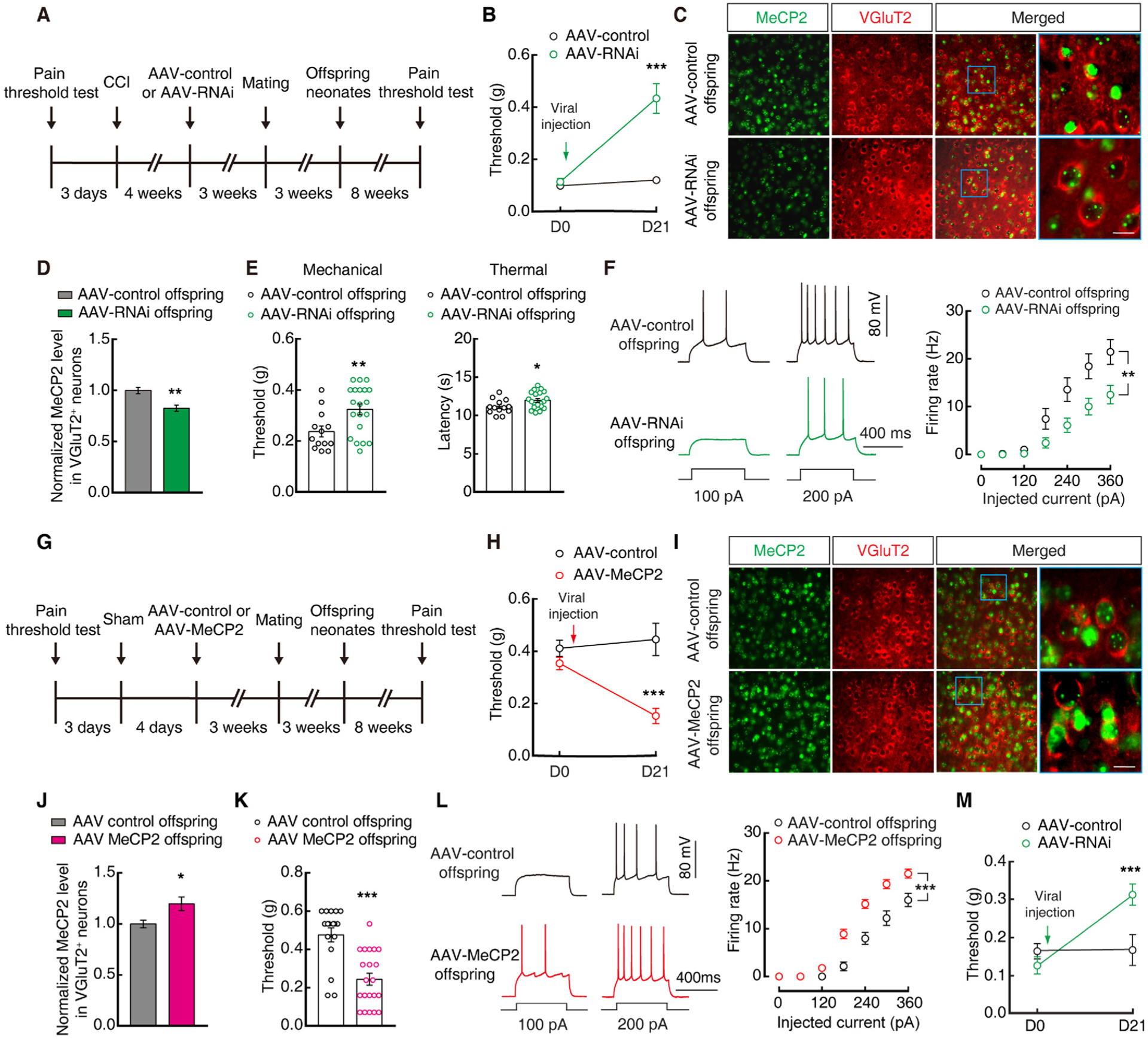
Maternal Glu^S1L2/3^ neuronal MeCP2 controls pain hypersensitivity in offspring. (A) Experimental timeline of breeding scheme. (B) Pain threshold of maternal mice with S1^L2/3^ infusion of AAV-control or AAV-RNAi. (C and D) Fluorescence images of MeCP2 expression (C) and statistical data (D) in Glu ^S1L2/3^ neurons in offspring from CCI maternal mice with S1^L2/3^ infusion of AAV-control or AAV-RNAi. Scale bar: 10 μm. (E and F) Pain threshold (E) and Glu^S1L2/3^ neuronal action potential firings (F) in offspring from CCI maternal mice with S1^L2/3^ infusion of AAV-control or AAV-RNAi. (G) Experimental timeline of breeding scheme. (H) Pain threshold of maternal mice with S1^L2/3^ infusion of AAV-MeCP2 or AAV-control. (I and J) Fluorescent images of MeCP2 expression (I) and statistical data (J) in Glu^S1L2/3^ neurons in offspring from sham maternal mice with S1^L2/3^ infusion of AAV-control or AAV-MeCP2. Scale bar: 10 μm. (K and L) Pain threshold (K) and Glu^S1L2/3^ neuronal action potential firings (L) in offspring from sham maternal mice with S1^L2/3^ infusion of AAV-control or AAV-MeCP2. (M) Pain threshold of offspring with S1^L2/3^ infusion of AAV-control or AAV-RNAi. These offspring were from sham maternal mice with S1^L2/3^ infusion of AAV-MeCP2. The blue boxes depict the area shown in the boxes of the S1^L2/3^. Data are presented as mean ± SEM. For statistical analyses, see Supplementary Table 9. *P < 0.05, **P < 0.01, ***P < 0.001.

Next, we examined whether MeCP2 contributed to pain transgenerational transmission. Similarly, in maternal mice, CCI-induced pain sensitization was inhibited by S1^L2/3^ infusion of AAV-RNAi (Fig. 5A, B). Strikingly, the S1^L2/3^ neuronal MeCP2 protein level decreased in their offspring (Fig. 5C, D); these offspring displayed increased pain threshold (Fig. 5E) and decreased Glu^S1^ neuronal activity when compared with that of the offspring from CCI mice with S1^L2/3^ infusion of the AAV-control (Fig. 5F). In addition, in sham maternal mice, overexpression of Glu^S1L2/3^ neuronal MeCP2 by S1^L2/3^ infusion of AAV-MeCP2 reduced pain threshold (Fig. 5G, H); in particular, their offspring displayed significant pain sensitization with increased Glu^S1L2/3^ neuronal MeCP2 level and neuronal activity (Fig. 5I–L). Notably, this pain sensitization was restored by S1^L2/3^ injection of AAV-RNAi in the offspring (Fig. 5M). These results suggest that MeCP2 contributes to pain transgenerational transmission, which is likely *via* regulation of Glu^S1L2/3^ neuronal activity.

### 3.5. MeCP2-mediated gene transcriptional activity in pain sensitization

**Fig. 6.**
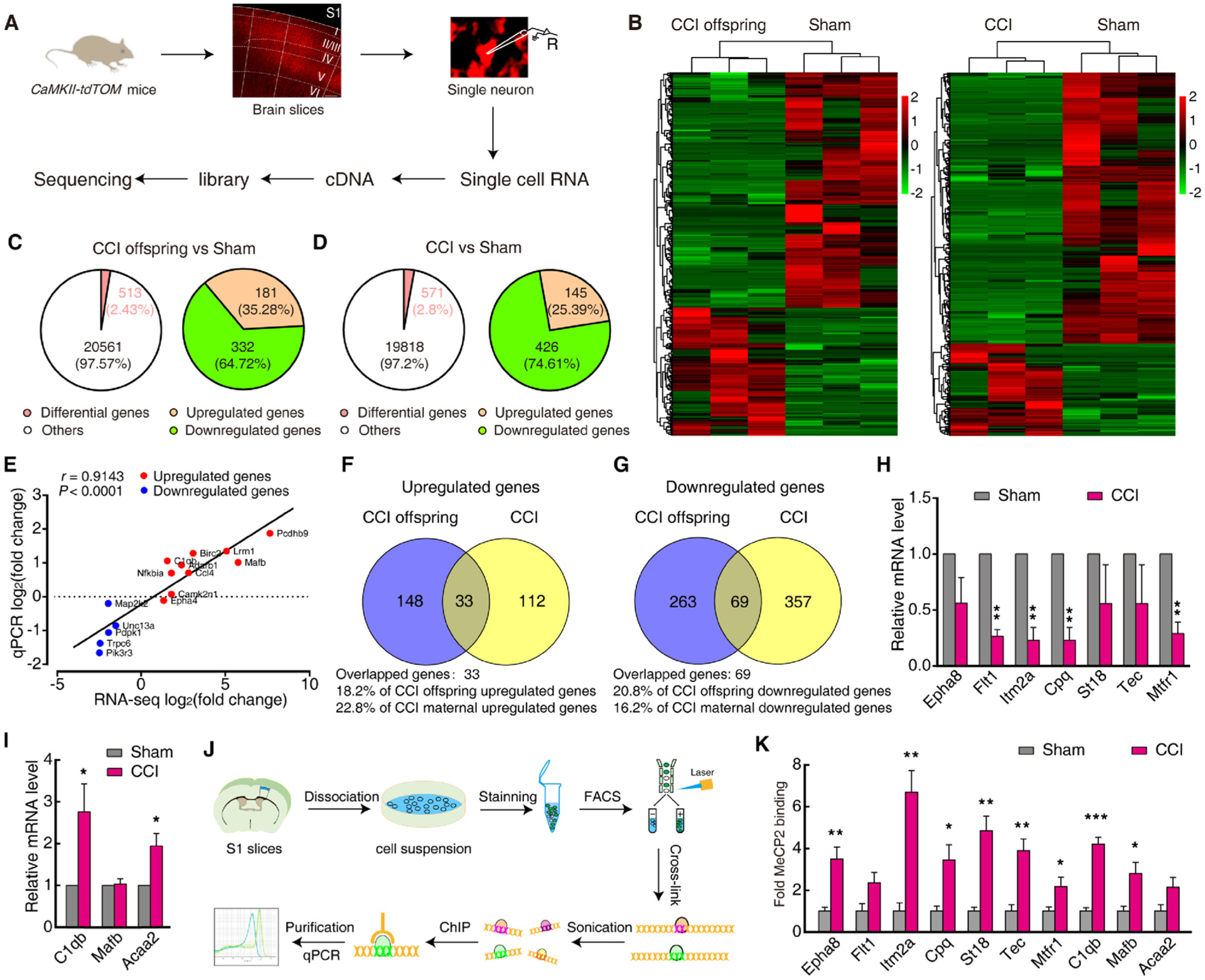
MeCP2-mediated gene transcriptional activity in pain sensitized offspring. (A) Workflow diagram depicting rapid isolation of a single Glu^S1L2/3^ neuron for single-cell RNA-seq. (B) Expression levels are depicted according to the color scale at the right. (C and D) Left: The relative percentage of differentially expressed genes in CCI offspring (C) and CCI maternal mice (D) compared with sham mice; Right: The proportion of upregulated and downregulated genes among the total differentially expressed genes in the left. (E) Further validation of RNA-seq for selected genes in (C) was conducted by real-time PCR from Glu^S1L2/3^ neurons sorted by FACS. Plotted here are the ranked log2 fold changes of 15 genes obtained from the RNA-seq *versus* those obtained from the qRT-PCR. They were correlated indicated by Spearman’s rank correlation coefficient. Red and blue colors indicate increased and decreased gene expression in RNA-seq, individually. (F and G) Overlap of upregulated (F) and downregulated (G) genes in CCI maternal mice and their offspring. (H and I) qRT-PCR confirming the expression of selected genes in (D) from Glu^S1L2/3^ neurons sorted by FACS (*n* = 4). Data are presented as the relative fold difference from sham control mice. (J) Workflow diagram depicting FACS protocol to dissociate and sort Glu^S1L2/3^ neurons for ChIP experiments. (K) Levels of MeCP2 on selected genes (in H and I) promoters. Data are presented as mean ± SEM. For statistical analyses, see Supplementary Table 9. * *P* < 0.05, ** *P* < 0.01, ***P < 0.001.

We next examined gene expression profiles in maternal mice and their offspring. A single-cell technique with high-coverage RNA-seq, which enables a better understanding of a certain cell’s transcriptome (Fuzik et al., 2016; Li et al., 2016), was performed on a S1^L2/3^ neuron. Under a microscope, a CaMKII-tdTOM neuron was aspirated with glass pipettes for single-cell amplification and sequencing (Fig. 6A). Following analysis, 12,911 of the total detected 21,685 genes commonly existed in Glu^S1L2/3^ neurons among CCI and sham mice, and their offspring using a single-cell technique with high-coverage RNA-seq (Supplementary Fig. 7A). The profile of gene expression was significantly changed in CCI maternal and their offspring Glu^S1L2/3^ neurons (Fig. 6B). We found that 513 genes were misregulated in CCI offspring when compared with sham (≈2.43 %), of which 181 genes were upregulated and 332 genes were downregulated (Fig. 6C and Supplementary Table. 2–3). These differential genes were related to multiple molecular functions, such as immune response and action potential firing, in Gene Ontology (GO) terms (Supplementary Fig. 7B–E). Further validation of RNA-seq was conducted using real-time PCR for a randomly selected 15 genes in Glu^S1L2/3^ neurons sorted by fluorescence-activated cell sorting (FACS) (Supplementary Fig. 8). These quantifications of gene expression correlated well with the original RNA-seq (Fig. 6E). In addition, in CCI maternal mice, 571 genes were misregulated (145 upregulated and 426 downregulated) when compared with those of sham control mice (≈2.8 %) (Fig. 6D, and Supplementary Table. 4–5). Notably, 102 differentially expressed genes (33 upregulated and 69 downregulated) were identified in both CCI offspring and their maternal mice, and the direction of variation was consistent (Fig. 6F, G and Supplementary Table. 6–7); a randomly selected 10 genes of these 102 genes were verified by real-time PCR in Glu^S1L2/3^ neurons sorted by FACS (Fig. 6H, I).

To determine whether these differentially expressed genes were regulated by MeCP2, we used chromatin immunoprecipitation (ChIP) to assess MeCP2 across the gene promoters in S1^L2/3^ neurons sorted by FACS (Fig. 6J). We selected the 10 genes in Fig. 6F and 6 G as targets. We found that the level of MeCP2 binding to eight gene promoters, including *Eph receptor A8 (Epha8), integral membrane protein 2A (Itm2a), carboxypeptidase Q (Cpq), tec protein tyrosine kinase (Tec), suppression of tumorigenicity 18 (St18), mitochondrial fission regulator 1 (Mtfr1), complement component 1, q subcomponent, beta polypeptide (C1qb)*, and *v-maf musculoaponeurotic fibrosarcoma oncogene family, protein B (Mafb)*, were significantly increased in CCI maternal mice when compared with that of sham mice (Fig. 6K).

## 4. Discussion and conclusions

This study demonstrates MeCP2-mediated fine-control of neuronal maladaptive plasticity through which pain transgenerational transmission is generated. Central to this process is a molecular mechanism involving the upregulation of MeCP2 in Glu^S1L2/3^ neurons that increases neuronal activity in maternal mice under chronic pain conditions. These epigenetic tags are passed to the next generation, leading to pain sensitization in offspring. Continued efforts to determine how and when epigenetic-regulated gene function influences chronic pain phenotypes will provide insights into genetics-based pain diagnoses and preventive treatments, and individual-based pain management in terms of ‘heri-table’ non-genetic traits.

Increased risk of having pain in offspring of parents with chronic pain is suggestive of pain transgenerational transmission (Higgins et al., 2015; Hoftun et al., 2013; Klengel et al., 2016). Consistent with this notion, we found 35 of 54 offspring suffered from pain from 54 parents with chronic low back pain. Although this sample was limited, the rate (≈65 %) was higher than the reported pain heritability of 16–50 % (Hakim et al., 2003; Smith et al., 2011). The reason is likely due to different pain intensities in the experimental participants. In this study, the pain subjects, who have to be hospitalized, were recruited from a pain clinic, suffering from severe pain (more than five years). This may lead to the pain outcome being greater than those used in large-scale epidemiological studies. In animals, the offspring from maternal mice with chronic pain, but not paternal mice, displayed significant pain sensitization. This is consistent with human studies in that the prevalence of chronic pain was higher in women than in men (Bouhassira et al., 2008). Previous studies have found that the associations between parental chronic pain and psychological symptoms in children may depend on the sex and age of the child as well as parental sex (Kaasboll et al., 2012). For example, mothers with chronic pain report more physical and psychological problems than children of fathers with chronic pain (Higgins et al., 2015; Kaasboll et al., 2012), and girls seem to be more vulnerable to the influence of maternal pain (Stone and Wilson, 2016). This proposes that paternal germline effects, which could be produced under persistent pain conditions presented in the current study, are usually overridden by maternal effects (Curley et al., 2011). Thus, females are predicted to dynamically adjust their reproductive investment in response to environmental factors, with consequences for offspring brain and behavioral development (Curley et al., 2011). In particular, this pain phenotype was also exhibited in the second generation, suggesting that this effect could be termed a transgenerational transmission (Daxinger and Whitelaw, 2012; Grossniklaus et al., 2013). Due to the limited experimental amenability of studies in humans, this mouse model can provide a new platform to investigate the potential mechanism underlying the transgenerational transmission of chronic pain.

The primary somatosensory cortex, as a critical brain site in pain signal processing, is activated in patients with multiple types of chronic pain (Campbell and Meyer, 2006; Costigan et al., 2009; Vierck et al., 2013). In our human fMRI study, S1 was activated in chronic pain offspring as well, suggesting a shared brain area in both offspring and parental pathological pain. On a cellular level, the adaptive Glu^S1L2/3^ neuronal hyperactivity programmed by the chronic pain maternal lineage appears to be imprinted in their offspring. This could lead to spontaneous pain or sensitization to pain environments, such as inflammation and neural injury, in offspring. In addition, pain has been considered as an inherent stressor. It has been shown that maternal stress experience is a risk factor for fetal brain development, which often exhibits a sex bias (Jasarevic et al., 2018; Kim et al., 2017; Shin Yim et al., 2017). In this aspect, the molecular mechanism has been extensively investigated, but is still poorly understood. The evidence from the current study suggests that maternal Glu^S1L2/3^ neuronal im-printing reprogrammed by chronic pain is passed to the offspring and correlates to the pain phenotype.

Mutation-caused pain hypersensitivity is quite rare, which is incompatible with the high rate of pain in offspring (Crow et al., 2013; Diatchenko et al., 2007). Hence, we propose a hypothesis for an epigenetic mechanism of pain transgenerational transmission. MeCP2, as a transcriptional repressor or activator by recognizing epigenetic states (e.g., methylated DNA and chromatin conformation), plays a crucial role in the underlying pathogenesis of chronic pain (Chahrour et al., 2008; Na and Monteggia, 2011). A previous study has shown that 75 % of MeCP2-caused Rett Syndrome patients have an abnormal pain response (Downs et al., 2010). In animal pain models, the current study also demonstrates that Glu^S1L2/3^ neuronal MeCP2 is both necessary and sufficient for the transgenerational transmission of pain. Our data have not yet supported a germline passage of epigenetic modification. It is equally possible that maternal chronic pain may change the development of the female offspring, leading to chronic pain manifestation; since the offspring have chronic pain, their chronic pain factors can again pass to the next generation. This could mimic chronic visceral pain that develops developmental after neonatal insults, except that such insults are delivered from mothers with chronic pain. This can also explain why paternal factors have less impact on offspring.

Given that dysfunction of MeCP2 alters synaptic plasticity, an immediate question is which and how these genes were involved. We found that 102 genes in the Glu^S1L2/3^ neuronal transcriptional profiles are simultaneously changed in both maternal and offspring mice with pain compared with their controls. This suggests that maternal chronic pain would more likely affect transcriptional patterns, rather than a single candidate gene. This raises the possibility that these pain-related genes for pain regulation were conserved, and their dysfunction would be passed to offspring. Although the role of these genes was not extensively investigated in the current study, previous studies have shown that many of these genes such as the typical pain-related genes *C1qb*, *Tec*, *Cpq*, *Epha* and *Mafb* modulate synaptic plasticity and pain behaviors (Tozaki-Saitoh et al., 2018; Vasileiou et al., 2013; Wang et al., 2009; Yang et al., 2018; Zorina-Lichtenwalter et al., 2016). Among these genes, the MeCP2 binding pattern on their promoters is changed under persistent pain conditions. It is generally thought that for most genes, an epigenetic “clean slate” is started after conception by total reprogramming, whereas a small minority of genes possesses epigenetic tags, such as DNA methylation and histone modification, surviving the reprogramming process (Kanherkar et al., 2014). Thus, the maternal germline epigenetic changes on a population of gene transcriptional zones have been proposed to be possibly inherited in the F2 generation in multiple species (Grossniklaus et al., 2013; Heard and Martienssen, 2014). Supporting this notion, our data from single-cell RNA-seq and ChIP further demonstrate the roles of these MeCP2-medited pain-related genes. Certainly, we cannot exclude the importance of a single gene in the transgenerational transmission of pain, such as the *MeCP2* gene itself.

## Supplementary Material

Supplementary Files

Supplementary Video 1

Supplementary Video 2
