# Supplementary material for: MeCP2 mediates transgenerational transmission of chronic pain
Source: Prog Neurobiol. Author manuscript; Available in PMC 2021 Aug 16. (PMC8367090; doi:10.1016/j.pneurobio.2020.101790)
Supplement: Supplementary Files [file NIHMS1722455-supplement-Supplementary_Files.pdf]

Supplementary Figures

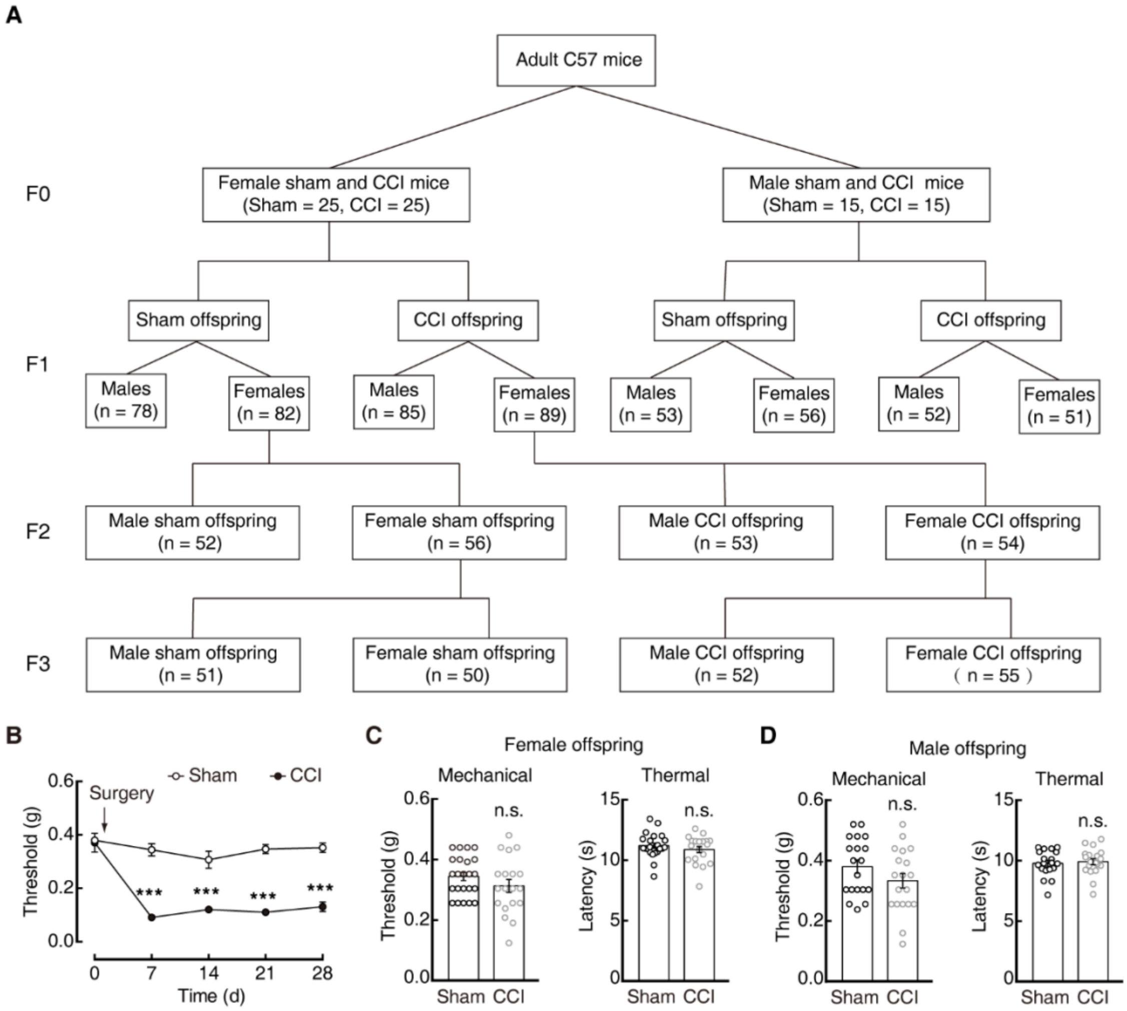

Supplementary Fig. S1 *Breeding scheme and pain behavior*. (A) Scheme of breeding paradigms. (B) Time course of CCI-induced sensory pain in male mice. (C and D) The pain threshold of female (C), and male (D) offspring from sham and CCI paternal mice. Data are presented as mean ± SEM. For statistical analyses, see Supplementary Table 9. n.s., not significant; ***P < 0.001.

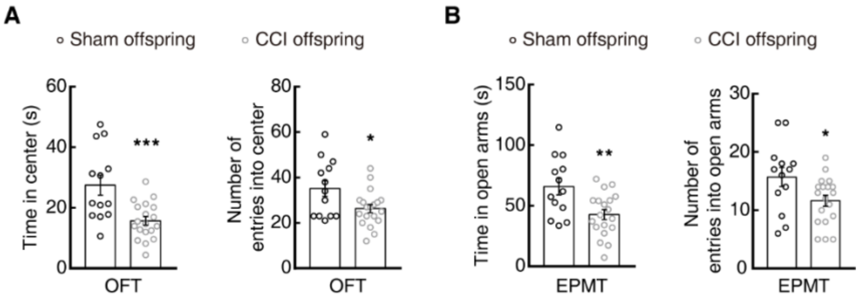

Supplementary Fig. S2 *Anxiety-like behavior of female offspring from CCI maternal mice*. (A and B) Performance of sham and CCI offspring mice in the open field test (OFT) (A) and elevated plus maze test (EPMT) (B). Data are presented as mean ± SEM. For statistical analyses, see Supplementary Table 9. *P < 0.05, **P < 0.01, ***P < 0.001.

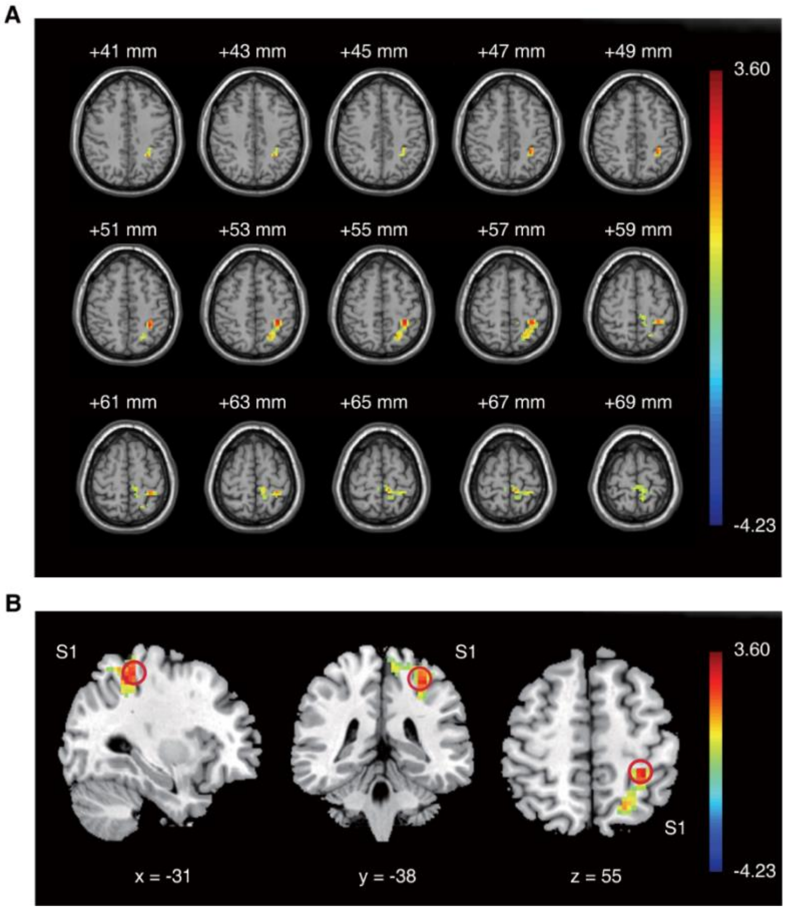

Supplementary Fig. S3 *Altered brain activity of offspring from parents with chronic pain*. (A) Human fMRI images showing brain areas with significant differences in offspring with pain compared with those without pain. These offspring are from parents with chronic pain. The results are shown as z-score maps. The number above each slice indicates the MNI z coordinate. (B) The red circle in each image represents the region containing the primary somatosensory cortex (S1) from the offspring indicated in (A). The color bars show the *t*-score to the right of the images. The data for each altered brain region are presented in the Table S1.

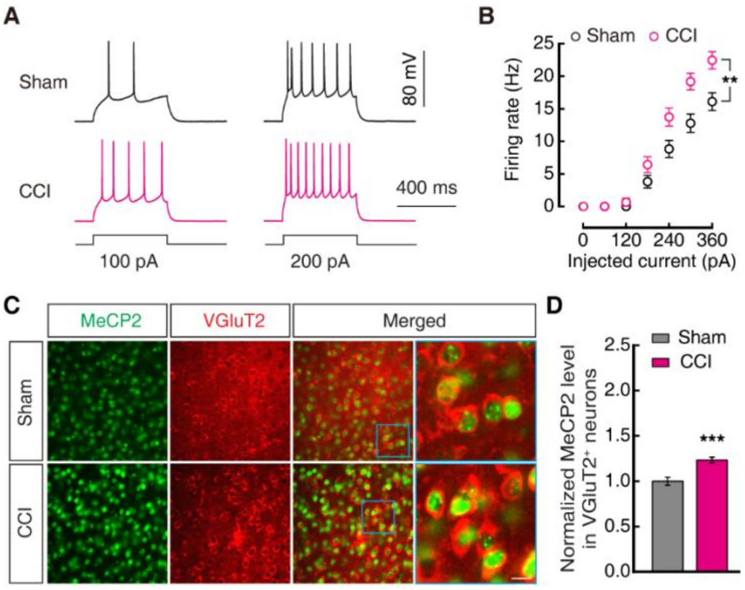

Supplementary Fig. S4 *Increased Glu$^{S1L2/3}$ neuronal activity and expression of MeCP2 protein in CCI maternal mice.* (A and B) Sample traces (A) and statistical data (B) of action potential firings recorded from S1$^{L2/3}$ tdTOM-expressing neurons in sham and CCI maternal mice. (C and D) Fluorescence images (C) and statistical data (D) of Glu$^{S1L2/3}$ neuronal MeCP2 in sham and CCI maternal mice. Levels of MeCP2 immunostaining were normalized to those of sham controls. Scale bar: 10 μm. The blue boxes depict the area shown in the boxes of the S1$^{L2/3}$. Data are presented as mean ± SEM. For statistical analyses, see Supplementary Table 9. $^{**}P<0.01$, $^{**}P < 0.001$.

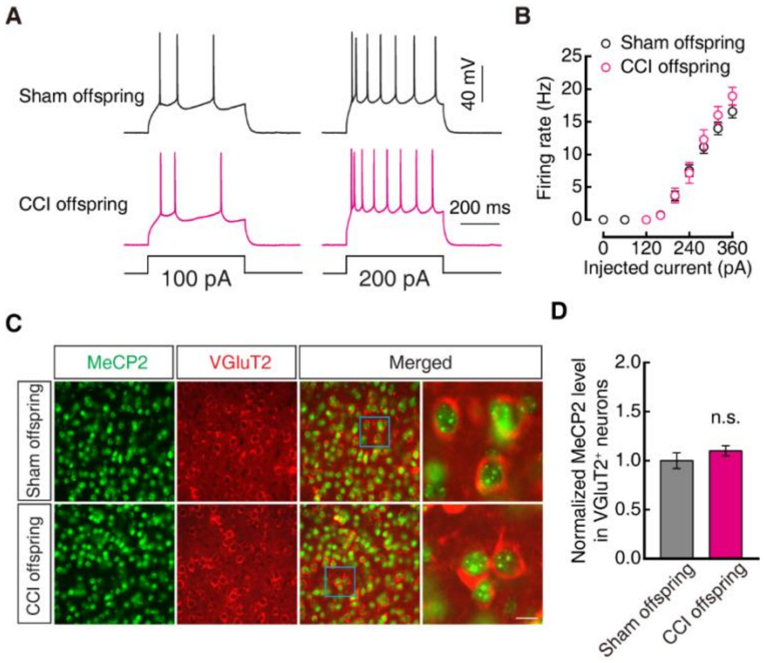

Supplementary Fig. S5. *Activity and MeCP2 expression of Glu$^{S1L2/3}$ neurons in male offspring.* (A and B) Sample traces (A) and statistical data (B) of action potential firings recorded from S1$^{L2/3}$ tdTOM-expressing neurons in sham and CCI male offspring from maternal mice. (C and D) Fluorescent images (C) and statistical data (D) of Glu$^{S1L2/3}$ neuronal MeCP2 in sham and CCI male offspring from maternal mice. Levels of MeCP2 immunostaining were normalized to those of sham controls. Scale bar: 10 µm. The blue boxes depict the area shown in the boxes of the S1$^{L2/3}$. Data are presented as mean ± SEM. For statistical analyses, see Supplementary Table 9. n.s., not significant.

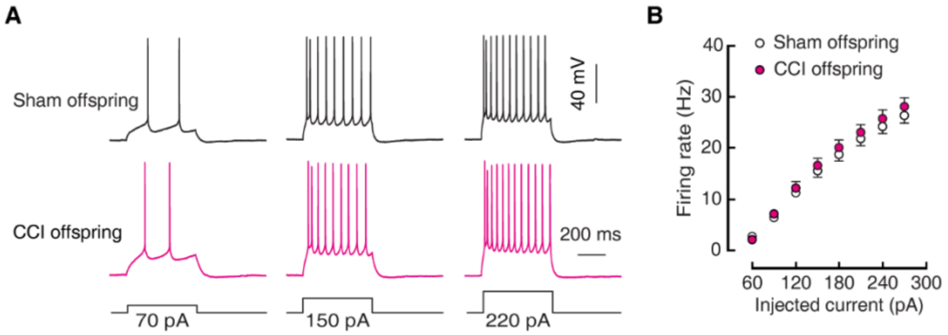

Supplementary Fig. S6. *Activity of Glu^{S1L5} neurons in female offspring.* (A and B) Sample traces (A) and statistical data (B) of action potential firings recorded from S1^{L5} tdTOM-expressing neurons in sham and CCI female offspring from maternal mice. Data are presented as mean ± SEM. For statistical analyses, see Supplementary Table 9.

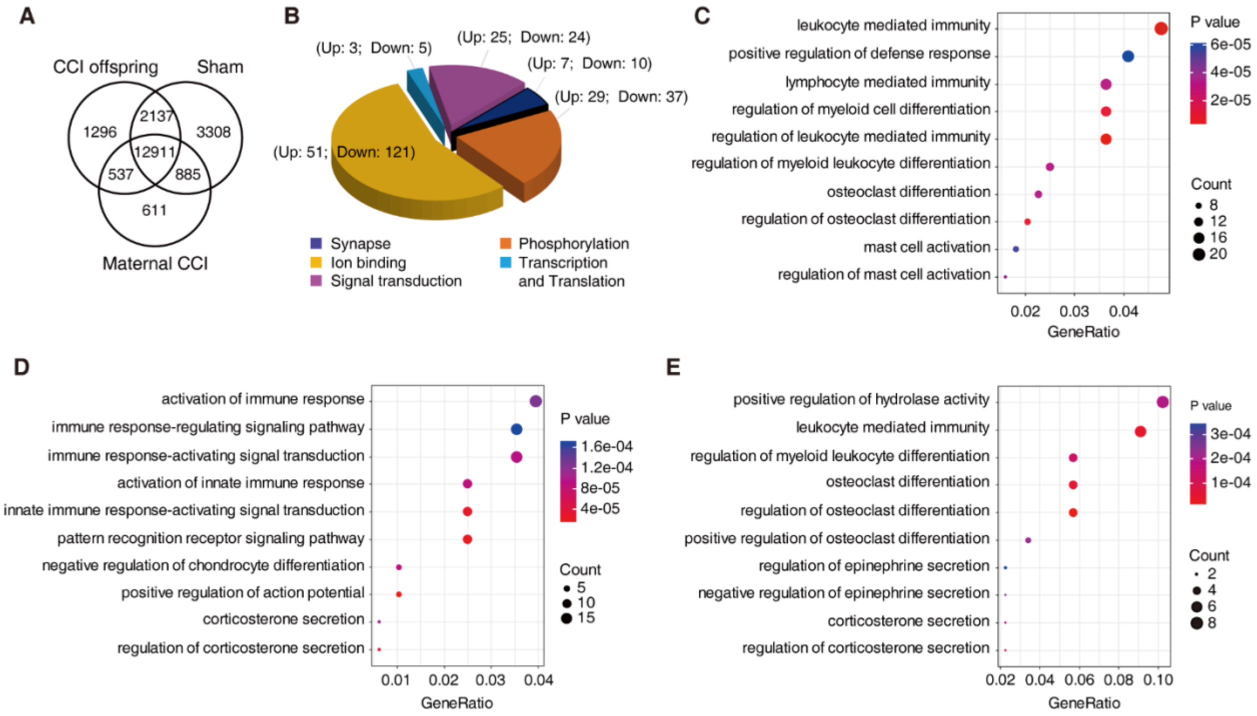

Supplementary Fig. S7 *Functional annotation of differentially expressed genes.* (A) Overlap of the detected genes by RNA-Seq in CCI offspring, as well as CCI maternal and sham control mice. (B) Functional annotation of the differential genes in CCI offspring compared with that of their maternal mice according to Gene Ontology (GO) terms. (C–E) The GO–enrichment analysis for the biological processes of differentially expressed genes in CCI offspring (C**,** 440 genes), CCI maternal (D, 481 genes), and overlapped differential genes (E, 88 genes). Each symbol represents a GO term (denoted in plot); color indicates unadjusted *P* value (significance of the GO term; top key), and symbol size is proportional to the number of genes (bottom key).

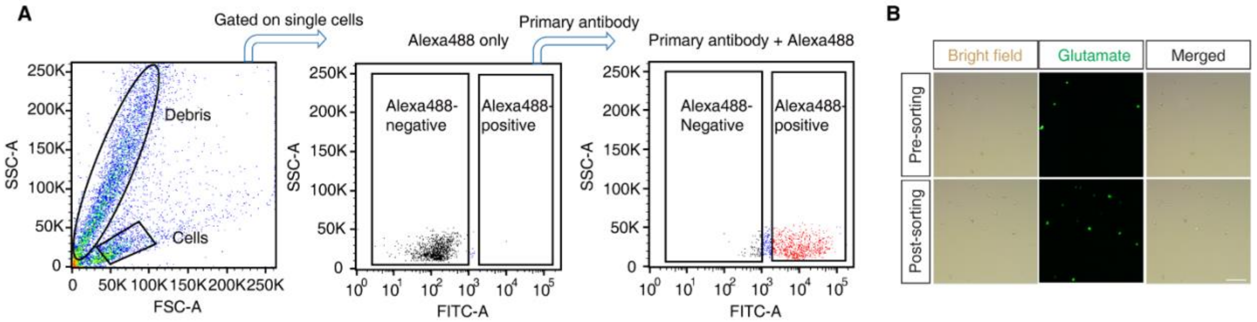

Supplementary Fig. S8 *Glu$^{S1L2/3}$ neurons sorted by FACS.* (A) Representative flow cytometric data, regions and sort gate. FSC-A (forward scatter-area) versus SSC-A (side scatter-area) plots were used to identify cells of interest based on size and granularity (complexity). FITC-A (fluorescein isothiocyanate-area) versus SSC-A plots were used to separate Alexa488 (FITC)-specific cells from fluorescent events. (B) Imaging of Glu$^{S1L2/3}$ neurons before and after FACS. FACS-sorted cells remained morphologically similar to presorted cells, and were 100% Alexa Fluor 488^{+} cells. Scale bar, 50 μm.

Supplementary Movie 1. Shock-evoked calcium activity in S1 neurons of sham offspring. The duration of shock stimulation is indicated by “Tail shock.”

Supplementary Movie 2. Shock-evoked calcium activity in S1 neurons of CCI offspring. The duration of shock stimulation is indicated by “Tail shock.”

Supplementary Table 1. Brain areas with altered ALFF in offspring with pain versus offspring without pain. The offspring are from parents with chronic pain.

Brain regions	Hemisphere	Voxel number
Superior parietal lobule	Left	56
Postcentral gyrus	Left	49
Inferior parietal lobule	Left	46
Paracentral lobule	Left	27
Paracentral lobule	Right	7

Supplementary Table 2. The list of genes (181) that are upregulated in $Glu^{S1L2/3}$ neurons of CCI offspring relative to those of sham controls (fold change ≥ 2, $P < 0.05$).

Ensembl Gene ID	Gene Symbol	Log2 Fold Change (CCI offspring over sham)	P value (CCI offspring over sham)
ENSMUSG00000075528	Aarsd1	6.662321	3.86E-05
ENSMUSG00000038393	Txnip	+Inf	6.57E-05
ENSMUSG00000008226	Scrn3	7.222565	0.000252
ENSMUSG00000034116	Vav1	7.44114	0.000346
ENSMUSG00000018415	Gid4	+Inf	0.000519
ENSMUSG00000024663	Rab3il1	+Inf	0.000708
ENSMUSG00000049600	Zbtb45	+Inf	0.000789
ENSMUSG00000022817	Itgb5	7.476424	0.000952
ENSMUSG00000054675	Tmem119	3.901824	0.001036
ENSMUSG00000018189	Uchl5	3.224762	0.001314
ENSMUSG00000026841	Fibcd1	+Inf	0.001373
ENSMUSG00000041515	Irf8	+Inf	0.001243
ENSMUSG00000042246	Tmc7	+Inf	0.001316
ENSMUSG00000036880	Acaa2	+Inf	0.001423
ENSMUSG00000000562	Adora3	7.817621	0.041858
ENSMUSG00000000567	Sox9	1.715953	0.033455
ENSMUSG00000001768	Rin2	6.146519	0.024435
ENSMUSG00000001774	Chordc1	1.744117	0.032568
ENSMUSG00000002147	Stat6	+Inf	0.039887
ENSMUSG00000002731	Prkra	4.373376	0.049992
ENSMUSG00000003974	Grm3	5.080049	0.045434
ENSMUSG00000004837	Grap	6.100972	0.043699
ENSMUSG00000005125	Ndrg1	5.255311	0.004731
ENSMUSG00000005204	Senp3	3.24811	0.012146
ENSMUSG00000005882	Uqcc1	1.60841	0.048461
ENSMUSG00000006611	Hfe	8.72557	0.007207
ENSMUSG00000007891	Ctsd	1.552807	0.01919
ENSMUSG00000010025	Aldh3a2	2.539602	0.022506

ENSMUSG00000013523	Bcas1	+Inf	0.015421
ENSMUSG00000015711	Prune	3.460104	0.034671
ENSMUSG00000015852	Fcrls	2.678449	0.037038
ENSMUSG00000016150	Tenm1	3.327553	0.047591
ENSMUSG00000017466	Timp2	2.589272	0.011859
ENSMUSG00000018008	Cyth4	+Inf	0.005509
ENSMUSG00000018593	Sparc	1.828108	0.016703
ENSMUSG00000018740	Slc25a35	6.060934	0.007245
ENSMUSG00000018930	Ccl4	2.812417	0.005207
ENSMUSG00000019773	Fbxo5	+Inf	0.020259
ENSMUSG00000020029	Nudt4	2.3523	0.016542
ENSMUSG00000020262	Adarb1	2.398404	0.042218
ENSMUSG00000020381	3010026O09Rik	6.590065	0.028072
ENSMUSG00000020495	Smg8	+Inf	0.02525
ENSMUSG00000020630	Rnaseh1	+Inf	0.001823
ENSMUSG00000020639	Pfn4	5.568987	0.027037
ENSMUSG00000021009	Ptpn21	4.605004	0.047244
ENSMUSG00000021025	Nfkbia	1.798796	0.030757
ENSMUSG00000021215	Net1	2.897692	0.037494
ENSMUSG00000021234	Fam161b	4.797957	0.048376
ENSMUSG00000021238	Aldh6a1	4.763849	0.005856
ENSMUSG00000021245	Mlh3	+Inf	0.005138
ENSMUSG00000021532	Fastkd3	+Inf	0.03357
ENSMUSG00000021665	Hexb	2.026319	0.014637
ENSMUSG00000021721	Htr1a	+Inf	0.003664
ENSMUSG00000021948	Prkcd	+Inf	0.030167
ENSMUSG00000022020	Naa16	5.366654	0.030176
ENSMUSG00000022023	Wbp4	1.893105	0.026122
ENSMUSG00000022350	E430025E21Rik	2.227438	0.036404
ENSMUSG00000022500	Litaf	4.238796	0.028147
ENSMUSG00000022525	Hrasls	3.088661	0.031888
ENSMUSG00000024104	Fam21	2.107461	0.035726

ENSMUSG00000024165	Hn1l	+Inf	0.00826
ENSMUSG00000024507	Hsd17b4	3.055598	0.047327
ENSMUSG00000024621	Csf1r	2.765164	0.003987
ENSMUSG00000024679	Ms4a6d	7.195351	0.027548
ENSMUSG00000025130	P4hb	1.702041	0.022821
ENSMUSG00000026029	Casp8	8.131696	0.01429
ENSMUSG00000026235	Epha4	1.340353	0.0463
ENSMUSG00000026288	Inpp5d	3.511391	0.026258
ENSMUSG00000026303	Mlph	+Inf	0.02014
ENSMUSG00000026358	Rgs1	+Inf	0.004708
ENSMUSG00000026471	Mr1	+Inf	0.040752
ENSMUSG00000026679	Enkur	+Inf	0.049924
ENSMUSG00000027193	Api5	2.087663	0.009644
ENSMUSG00000027301	Oxt	5.343637	0.027477
ENSMUSG00000027339	Rassf2	4.793803	0.013111
ENSMUSG00000027355	Tmco5	7.38388	0.038066
ENSMUSG00000027589	Pcmtd2	2.243668	0.020044
ENSMUSG00000027708	Dcun1d1	2.308175	0.013542
ENSMUSG00000027848	Olfml3	2.28091	0.008642
ENSMUSG00000028476	Reck	5.39997	0.024301
ENSMUSG00000028581	Laptm5	1.617317	0.042678
ENSMUSG00000029119	Man2b2	+Inf	0.031667
ENSMUSG00000029366	Dck	2.236459	0.027008
ENSMUSG00000030036	Mogs	+Inf	0.003106
ENSMUSG00000031154	Otud5	3.649217	0.002296
ENSMUSG00000031197	Vbp1	2.040209	0.03322
ENSMUSG00000031210	Gpr165	+Inf	0.030076
ENSMUSG00000031284	Pak3	1.641323	0.020747
ENSMUSG00000031357	Syap1	4.492721	0.031763
ENSMUSG00000031605	Klhl2	1.824215	0.047484
ENSMUSG00000031843	Mphosph6	+Inf	0.013517
ENSMUSG00000031907	Zfp90	+Inf	0.021906

ENSMUSG00000031980	Agt	+Inf	0.017388
ENSMUSG00000032425	Zfp949	3.669982	0.005484
ENSMUSG00000032667	Pon2	3.671302	0.030996
ENSMUSG00000032878	Ccdc85a	2.833995	0.020438
ENSMUSG00000033111	3830406C13Rik	5.030267	0.02514
ENSMUSG00000034110	Kctd7	+Inf	0.030669
ENSMUSG00000034194	R3hcc1	5.546599	0.043105
ENSMUSG00000034453	Polr3b	5.416716	0.005238
ENSMUSG00000034487	Kdelc2	7.642995	0.036919
ENSMUSG00000034617	Mtrr	+Inf	0.040573
ENSMUSG00000034648	Lrrn1	5.056129	0.02614
ENSMUSG00000034652	Cd300a	7.847499	0.007842
ENSMUSG00000035601	Trmt10b	7.305427	0.009281
ENSMUSG00000035885	Cox8a	1.415088	0.037893
ENSMUSG00000036022	Fam122b	+Inf	0.007507
ENSMUSG00000036598	Ccdc113	+Inf	0.003967
ENSMUSG00000036854	Hspb6	+Inf	0.017849
ENSMUSG00000036887	C1qa	1.670257	0.017931
ENSMUSG00000036905	C1qb	1.558215	0.023217
ENSMUSG00000037151	Lrrc20	7.069288	0.035931
ENSMUSG00000037405	Icam1	4.740863	0.024562
ENSMUSG00000038048	Cntnap5c	+Inf	0.013746
ENSMUSG00000038147	Cd84	7.169971	0.030997
ENSMUSG00000038168	P3h2	6.004403	0.029071
ENSMUSG00000038605	Samd10	3.213228	0.013808
ENSMUSG00000038642	Ctss	2.026532	0.012661
ENSMUSG00000039850	Endov	+Inf	0.017188
ENSMUSG00000040033	Stat2	2.70888	0.027157
ENSMUSG00000040372	Gpr63	+Inf	0.036738
ENSMUSG00000040663	Clcf1	+Inf	0.024462
ENSMUSG00000040936	Ulk4	3.823108	0.02234
ENSMUSG00000041346	Wrap53	+Inf	0.001821

ENSMUSG00000041765	Ubac2	3.502225	0.039246
ENSMUSG00000041797	Abca9	+Inf	0.002899
ENSMUSG00000041992	Rapgef5	2.36407	0.03606
ENSMUSG00000042410	Agps	6.489273	0.031283
ENSMUSG00000043153	Ispd	+Inf	0.023687
ENSMUSG00000043262	Uevld	+Inf	0.012682
ENSMUSG00000043411	Usp48	1.591939	0.040183
ENSMUSG00000044071	Fam19a2	3.552689	0.025201
ENSMUSG00000044231	Nhlrc1	+Inf	0.036514
ENSMUSG00000044674	Fzd1	7.521591	0.012102
ENSMUSG00000044786	Zfp36	2.887224	0.01424
ENSMUSG00000045691	Thtpa	+Inf	0.001666
ENSMUSG00000045932	Ifit2	7.594421	0.012428
ENSMUSG00000046447	Camk2n1	1.796633	0.007494
ENSMUSG00000046470	Sox18	+Inf	0.033539
ENSMUSG00000046561	Arsj	+Inf	0.024129
ENSMUSG00000049164	Zfp518a	5.09986	0.04437
ENSMUSG00000050244	Heatr1	3.938943	0.011235
ENSMUSG00000050271	D8Ertd82e	1.839298	0.046567
ENSMUSG00000050567	Maml1	5.24949	0.023935
ENSMUSG00000050855	Zfp940	6.058753	0.024096
ENSMUSG00000050973	Gdpgp1	+Inf	0.025727
ENSMUSG00000051149	Adnp	+Inf	0.031806
ENSMUSG00000051242	Pcdhb9	7.618967	0.0339
ENSMUSG00000051537	Gm5124	+Inf	0.011353
ENSMUSG00000052395	Rft1	6.045968	0.04273
ENSMUSG00000052629	Gm9885	5.658066	0.040708
ENSMUSG00000052760	A630001G21Rik	+Inf	0.032739
ENSMUSG00000052821	Cysltr1	+Inf	0.033162
ENSMUSG00000052833	Sae1	2.261939	0.01925
ENSMUSG00000052848	C130026L21Rik	+Inf	0.013931
ENSMUSG00000053310	Nrgn	1.713964	0.024931

ENSMUSG00000054364	Rhob	1.927054	0.01826
ENSMUSG00000057367	Birc2	3.08204	0.04543
ENSMUSG00000060636	Rpl35a	6.296798	0.025287
ENSMUSG00000062127	Cttnbp2nl	+Inf	0.037836
ENSMUSG00000062175	Tgif2	+Inf	0.035676
ENSMUSG00000066324	Impad1	2.271866	0.016001
ENSMUSG00000068874	Selenbp1	+Inf	0.04411
ENSMUSG00000070737	Gm12942	+Inf	0.049769
ENSMUSG00000071652	Ints5	+Inf	0.018636
ENSMUSG00000073755	5730409E04Rik	3.566282	0.017202
ENSMUSG00000074622	Mafb	5.73856	0.012704
ENSMUSG00000074671	Tspyl3	7.611436	0.002016
ENSMUSG00000076617	Ighm	4.265268	0.01965
ENSMUSG00000078161	Erich3	6.851494	0.006937
ENSMUSG00000078906	Gm14444	+Inf	0.046083
ENSMUSG00000079227	Ccr5	+Inf	0.030479
ENSMUSG00000086181	C230034O21Rik	+Inf	0.03878
ENSMUSG00000086564	Cd101	+Inf	0.043049
ENSMUSG00000086873	Gm15672	+Inf	0.015855
ENSMUSG00000091387	Gcnt4	+Inf	0.019498
ENSMUSG00000096010	Hist4h4	+Inf	0.031619
ENSMUSG00000096687	AA474331	+Inf	0.001974
ENSMUSG00000102748	Pcdhgb2	8.418857	0.007533
ENSMUSG00000103049	Gm37311	+Inf	0.047487
ENSMUSG00000105460	Gm42671	7.351517	0.046928

76

Supplementary Table 3. The list of genes (332) that are downregulated in $Glu^{S1L2/3}$ neurons of CCI offspring relative to those of sham controls (fold change ≥ 2, *P* < 0.05).

Ensembl Gene ID	Gene Symbol	Log2 Fold Change (CCI offspring over sham)	P value (CCI offspring over sham)
ENSMUSG00000026224	4933407L21Rik	-9.60135	4.50E-05
ENSMUSG00000043207	Zmpste24	-7.41374	3.20E-05
ENSMUSG00000024664	Fads3	-Inf	0.000108
ENSMUSG00000030956	Fam53b	-3.74834	0.000104
ENSMUSG00000039474	Wfs1	-4.49233	0.000116
ENSMUSG00000027400	Pdyn	-Inf	0.000273
ENSMUSG00000042216	Sgsm1	-3.24306	0.000292
ENSMUSG00000044164	Rnf182	-Inf	0.000221
ENSMUSG00000103472	Pcdhga7	-8.1558	0.000367
ENSMUSG00000105302	Gm19817	-3.25889	0.000434
ENSMUSG00000004270	Lpcat3	-5.91985	0.000714
ENSMUSG00000029070	Mxra8	-Inf	0.001054
ENSMUSG00000030748	Il4ra	-9.77212	0.000959
ENSMUSG00000038260	Trpm4	-Inf	0.001099
ENSMUSG00000046191	Pcdhb20	-Inf	0.001131
ENSMUSG00000042185	Nfrkb	-3.44006	0.001332
ENSMUSG00000069539	Scyl2	-4.39023	0.001536
ENSMUSG00000000134	Tfe3	-2.26604	0.038756
ENSMUSG00000000420	Galnt1	-4.54961	0.026294
ENSMUSG00000001128	Cfp	-Inf	0.035912
ENSMUSG00000001767	Crnkl1	-Inf	0.011885
ENSMUSG00000001785	Pwp1	-4.68372	0.019331
ENSMUSG00000002957	Ap2a2	-1.81288	0.044261
ENSMUSG00000003752	Itpkc	-6.363	0.003003
ENSMUSG00000004462	Tbccd1	-Inf	0.019509
ENSMUSG00000004530	Coro1c	-1.69287	0.034922
ENSMUSG00000005338	Cadm3	-2.03455	0.023267
ENSMUSG00000005410	Mcm5	-Inf	0.023886

ENSMUSG00000005615	Pcyt1a	-3.51018	0.006532
ENSMUSG00000006127	Inpp5k	-2.05188	0.043798
ENSMUSG00000006763	Saal1	-8.46406	0.005801
ENSMUSG00000007379	Dennd2c	-Inf	0.044629
ENSMUSG00000007646	Rad51c	-Inf	0.011507
ENSMUSG00000008129	4930432K21Rik	-Inf	0.047162
ENSMUSG00000008307	1700109H08Rik	-Inf	0.013425
ENSMUSG00000008604	Ubqln4	-3.05149	0.027485
ENSMUSG00000008734	Gprc5b	-Inf	0.043546
ENSMUSG00000014778	Fhod1	-Inf	0.026031
ENSMUSG00000014782	Plekhg4	-Inf	0.021766
ENSMUSG00000015027	Galns	-Inf	0.043602
ENSMUSG00000015214	Mtmr1	-4.32879	0.027553
ENSMUSG00000016200	Syt14	-3.52128	0.021364
ENSMUSG00000016624	Phf21b	-Inf	0.021611
ENSMUSG00000016918	Sulf1	-Inf	0.025951
ENSMUSG00000017491	Rarb	-Inf	0.016184
ENSMUSG00000018363	Smurf2	-4.26727	0.048752
ENSMUSG00000018634	Crhr1	-3.24511	0.045937
ENSMUSG00000018800	Abca5	-1.56877	0.040136
ENSMUSG00000018841	Rad51d	-Inf	0.03023
ENSMUSG00000019338	Zfp687	-3.31449	0.040763
ENSMUSG00000019577	Pdk4	-Inf	0.02294
ENSMUSG00000019737	Syne4	-5.73852	0.009658
ENSMUSG00000019832	Rab32	-Inf	0.018882
ENSMUSG00000020134	Peli1	-1.71949	0.042027
ENSMUSG00000020364	Zfp354a	-Inf	0.003539
ENSMUSG00000020919	Stat5b	-3.79685	0.02658
ENSMUSG00000020953	Coch	-5.37191	0.008106
ENSMUSG00000021140	Pcnx	-2.49994	0.005777
ENSMUSG00000021492	F12	-Inf	0.036702
ENSMUSG00000021936	Mapk8	-1.43501	0.049834

ENSMUSG00000022008	Gpalpp1	-6.29355	0.016394
ENSMUSG00000022096	Hr	-5.14164	0.03332
ENSMUSG00000022262	Dnah5	-Inf	0.024753
ENSMUSG00000022292	Rrm2b	-3.05579	0.029556
ENSMUSG00000022325	Pop1	-Inf	0.047304
ENSMUSG00000022401	Xpnpep3	-Inf	0.007828
ENSMUSG00000022545	Ercc4	-3.2929	0.017088
ENSMUSG00000022568	Scrib	-Inf	0.024019
ENSMUSG00000022797	Tfrc	-1.8037	0.014984
ENSMUSG00000022833	Ccdc14	-Inf	0.032601
ENSMUSG00000022894	Adamts5	-Inf	0.013695
ENSMUSG00000022987	Zfp641	-Inf	0.042813
ENSMUSG00000023232	Serinc2	-Inf	0.003205
ENSMUSG00000024122	Pdpk1	-1.92233	0.04277
ENSMUSG00000024143	Rhoq	-3.52665	0.003269
ENSMUSG00000024505	Dtwd2	-Inf	0.033356
ENSMUSG00000024642	Tle4	-4.4459	0.014548
ENSMUSG00000024949	Sf1	-1.8384	0.034094
ENSMUSG00000025078	Nhlrc2	-4.93701	0.01804
ENSMUSG00000025085	Ablim1	-1.61061	0.041765
ENSMUSG00000025192	Entpd7	-3.40306	0.015293
ENSMUSG00000025395	Prim1	-Inf	0.00368
ENSMUSG00000025432	Avil	-Inf	0.04796
ENSMUSG00000025892	Gria4	-2.20221	0.036172
ENSMUSG00000025995	Wdr75	-2.91462	0.043133
ENSMUSG00000026039	Sgol2a	-Inf	0.022327
ENSMUSG00000026094	Stk17b	-Inf	0.003571
ENSMUSG00000026121	Sema4c	-Inf	0.043855
ENSMUSG00000026198	Abcb6	-3.86472	0.012323
ENSMUSG00000026280	Atg4b	-1.90425	0.031487
ENSMUSG00000026383	Epb41l5	-4.99578	0.047484
ENSMUSG00000026404	Ddx59	-Inf	0.006812

ENSMUSG00000026663	Atf6	-2.26071	0.00292
ENSMUSG00000027164	Traf6	-3.55786	0.01719
ENSMUSG00000027247	Arhgap1	-2.04618	0.019298
ENSMUSG00000027520	Zdbf2	-3.93561	0.032608
ENSMUSG00000027601	Mtfr1	-Inf	0.001801
ENSMUSG00000027739	Rab33b	-6.12685	0.00393
ENSMUSG00000027845	Dclre1b	-Inf	0.04016
ENSMUSG00000027965	Olfm3	-Inf	0.028273
ENSMUSG00000028080	Lrba	-4.33066	0.003515
ENSMUSG00000028164	Manba	-Inf	0.009303
ENSMUSG00000028600	Podn	-Inf	0.042458
ENSMUSG00000028661	Epha8	-Inf	0.008108
ENSMUSG00000028664	Ephb2	-4.55365	0.031438
ENSMUSG00000028698	Pik3r3	-2.46446	0.020648
ENSMUSG00000028838	Extl1	-1.85365	0.026084
ENSMUSG00000028978	Nos3	-Inf	0.049246
ENSMUSG00000029217	Tec	-Inf	0.016049
ENSMUSG00000029246	Ppat	-2.0407	0.034179
ENSMUSG00000029456	Acad10	-Inf	0.015839
ENSMUSG00000029505	Ep400	-1.6997	0.026714
ENSMUSG00000029648	Flt1	-Inf	0.014145
ENSMUSG00000029729	Zkscan1	-3.62909	0.027305
ENSMUSG00000030203	Dusp16	-4.62525	0.031987
ENSMUSG00000030313	Dennd5b	-1.85255	0.00982
ENSMUSG00000030409	Dmpk	-Inf	0.025236
ENSMUSG00000030505	Prmt3	-5.01675	0.044979
ENSMUSG00000030823	9130019O22Rik	-7.2128	0.017983
ENSMUSG00000030978	Rrm1	-8.57308	0.001974
ENSMUSG00000031239	Itm2a	-Inf	0.015588
ENSMUSG00000031309	Rps6ka3	-3.28875	0.03754
ENSMUSG00000031327	Chic1	-2.23048	0.024669
ENSMUSG00000031574	Star	-Inf	0.002989

ENSMUSG00000031627	Irf2	-2.55551	0.03132
ENSMUSG00000031642	Sh3rf1	-4.94151	0.004776
ENSMUSG00000031706	Rfx1	-2.2506	0.043592
ENSMUSG00000031885	Cbfb	-5.53597	0.007868
ENSMUSG00000031979	Cog2	-2.32636	0.039811
ENSMUSG00000031983	2310022B05Rik	-Inf	0.030467
ENSMUSG00000031997	Trpc6	-2.42013	0.016916
ENSMUSG00000032109	Nlrx1	-Inf	0.04348
ENSMUSG00000032125	Robo4	-Inf	0.037209
ENSMUSG00000032252	Glce	-4.49305	0.009078
ENSMUSG00000032396	Dis3l	-3.529	0.047994
ENSMUSG00000032536	Trak1	-1.46863	0.046644
ENSMUSG00000032702	Kank1	-Inf	0.038033
ENSMUSG00000033083	Tbc1d4	-6.48084	0.009796
ENSMUSG00000033253	Szt2	-2.52797	0.048842
ENSMUSG00000033313	Fbxl8	-Inf	0.023821
ENSMUSG00000033319	Fem1c	-4.99821	0.028706
ENSMUSG00000033478	Fam160b1	-2.37411	0.007212
ENSMUSG00000033623	Pcgf3	-5.82736	0.012946
ENSMUSG00000033712	Ccar2	-2.38013	0.036765
ENSMUSG00000033740	St18	-Inf	0.020396
ENSMUSG00000033849	B3galt2	-1.85566	0.039493
ENSMUSG00000033871	Ppargc1b	-3.58477	0.01272
ENSMUSG00000033904	Ccp110	-3.67735	0.005664
ENSMUSG00000033943	Mga	-1.88032	0.027865
ENSMUSG00000033964	Zbtb41	-8.88434	0.010586
ENSMUSG00000034023	Fancd2	-5.4318	0.013724
ENSMUSG00000034112	Atp2c2	-Inf	0.022195
ENSMUSG00000034429	Zfp707	-8.13095	0.032879
ENSMUSG00000034612	Chst11	-6.2601	0.007517
ENSMUSG00000034639	Setmar	-Inf	0.017875
ENSMUSG00000034732	Pabpc5	-Inf	0.039932

ENSMUSG00000034799	Unc13a	-1.49704	0.046516
ENSMUSG00000034898	Filip1	-4.80391	0.026362
ENSMUSG00000034910	Pygo1	-5.06364	0.023311
ENSMUSG00000035007	Rundc1	-1.39519	0.043759
ENSMUSG00000035027	Map2k2	-1.93882	0.019549
ENSMUSG00000035045	Zc3h12b	-3.80433	0.005306
ENSMUSG00000035049	Rrp12	-3.24852	0.012364
ENSMUSG00000035284	Vps13c	-2.22125	0.034652
ENSMUSG00000035486	Plk5	-2.25007	0.0089
ENSMUSG00000035504	Reep6	-7.4806	0.023393
ENSMUSG00000035891	Cerk	-4.32491	0.024619
ENSMUSG00000036054	Sugp2	-2.13426	0.048317
ENSMUSG00000036106	Prr5	-Inf	0.028653
ENSMUSG00000036698	Ago2	-2.11405	0.02873
ENSMUSG00000036834	Plch1	-5.14819	0.006248
ENSMUSG00000036882	Arhgap33	-1.72552	0.029088
ENSMUSG00000037190	Cyb561d2	-Inf	0.007444
ENSMUSG00000037224	Zfyve28	-3.31264	0.044487
ENSMUSG00000037313	Tacc3	-Inf	0.00221
ENSMUSG00000037708	Spag6l	-Inf	0.014545
ENSMUSG00000037795	N4bp2	-3.56828	0.038549
ENSMUSG00000038074	Fkbp14	-4.77761	0.00958
ENSMUSG00000038214	Bend3	-Inf	0.008202
ENSMUSG00000038259	Gdf5	-5.43954	0.02143
ENSMUSG00000038387	Rras	-Inf	0.025214
ENSMUSG00000038518	Jarid2	-2.53017	0.038344
ENSMUSG00000038708	Golga4	-1.97287	0.025933
ENSMUSG00000038806	Sde2	-2.16243	0.018055
ENSMUSG00000038827	Fam206a	-3.6288	0.007045
ENSMUSG00000039007	Cpq	-Inf	0.008148
ENSMUSG00000039021	Ttc16	-Inf	0.014525
ENSMUSG00000039116	Adgrg6	-6.66638	0.044487

ENSMUSG00000039294	BC017643	-8.10874	0.033442
ENSMUSG00000040054	Baz2a	-1.98397	0.040613
ENSMUSG00000040339	Fam102b	-4.93276	0.004128
ENSMUSG00000040599	Mis12	-Inf	0.002378
ENSMUSG00000040715	Rsc1a1	-8.11794	0.040886
ENSMUSG00000040734	Ppp1r13l	-Inf	0.024872
ENSMUSG00000040865	Ino80d	-4.25825	0.004815
ENSMUSG00000041258	Zfp236	-2.36526	0.025272
ENSMUSG00000041301	Cftr	-Inf	0.038714
ENSMUSG00000041343	Ankrd42	-5.00319	0.003652
ENSMUSG00000041372	B4galnt3	-Inf	0.016676
ENSMUSG00000041592	Sdk2	-2.7901	0.044343
ENSMUSG00000041997	Tlk1	-2.58838	0.001949
ENSMUSG00000042251	Pm20d1	-Inf	0.035768
ENSMUSG00000042473	Tbc1d8b	-Inf	0.033979
ENSMUSG00000042502	Cd2bp2	-2.51275	0.016406
ENSMUSG00000042742	B630005N14Rik	-2.7861	0.004958
ENSMUSG00000043456	Zfp536	-6.25476	0.002646
ENSMUSG00000043631	Ecm2	-Inf	0.02466
ENSMUSG00000043639	Rbm20	-6.87616	0.037725
ENSMUSG00000043909	Trp53bp1	-1.40548	0.049934
ENSMUSG00000044043	Pcdhb14	-Inf	0.040666
ENSMUSG00000044072	Eml6	-2.51663	0.045984
ENSMUSG00000044229	Nxpe4	-Inf	0.0186
ENSMUSG00000044254	Pcsk9	-5.78472	0.048605
ENSMUSG00000044949	Ubtd2	-Inf	0.028328
ENSMUSG00000045064	Zc2hc1c	-Inf	0.043619
ENSMUSG00000045103	Dmd	-1.88825	0.046989
ENSMUSG00000045962	Wnk1	-1.49849	0.034742
ENSMUSG00000046157	Tmem229b	-Inf	0.025601
ENSMUSG00000046185	Zfp84	-4.60985	0.047781
ENSMUSG00000046410	Kcnk6	-Inf	0.027217

ENSMUSG00000046808	Atp10d	-Inf	0.037677
ENSMUSG00000047415	Gpr68	-Inf	0.00712
ENSMUSG00000048078	Tenm4	-1.65183	0.047041
ENSMUSG00000048232	Fbxo10	-4.11701	0.007779
ENSMUSG00000048264	Dip2c	-3.82712	0.006815
ENSMUSG00000048330	Ric3	-3.00182	0.04949
ENSMUSG00000048537	Phldb1	-3.39308	0.02433
ENSMUSG00000049252	Lrp1b	-1.52228	0.026278
ENSMUSG00000049265	Kcnk3	-5.37304	0.017401
ENSMUSG00000049608	Gpr55	-Inf	0.024089
ENSMUSG00000049796	Crh	-Inf	0.02327
ENSMUSG00000049804	Armcx4	-4.23396	0.046485
ENSMUSG00000050908	Tvp23a	-Inf	0.002624
ENSMUSG00000050910	Cdr2l	-Inf	0.016648
ENSMUSG00000051413	Plagl2	-Inf	0.002219
ENSMUSG00000051499	Zfp786	-Inf	0.048431
ENSMUSG00000051747	Ttn	-4.11141	0.007814
ENSMUSG00000052155	Acvr2a	-2.43938	0.016875
ENSMUSG00000052221	Ppp1r36	-Inf	0.020026
ENSMUSG00000052273	Dnah3	-Inf	0.035178
ENSMUSG00000052752	Traf7	-2.11385	0.045758
ENSMUSG00000053214	Gm9899	-Inf	0.009009
ENSMUSG00000053646	Plxnb1	-Inf	0.042693
ENSMUSG00000053730	Tmem39b	-Inf	0.031799
ENSMUSG00000053897	Slc39a8	-Inf	0.03699
ENSMUSG00000055866	Per2	-1.52798	0.044025
ENSMUSG00000058153	Sez6l	-1.44032	0.040266
ENSMUSG00000058298	Mcm9	-7.85815	0.015112
ENSMUSG00000059013	Sh2d3c	-4.18394	0.014336
ENSMUSG00000060716	Plekhh1	-Inf	0.041332
ENSMUSG00000061979	Wbscr16	-5.52639	0.031268
ENSMUSG00000062190	Lancl2	-2.16198	0.021428

ENSMUSG00000062545	Tlr12	-Inf	0.049453
ENSMUSG00000064037	Gpn1	-5.14727	0.004722
ENSMUSG00000068114	Ccdc134	-6.046	0.018027
ENSMUSG00000071477	Zfp777	-Inf	0.040587
ENSMUSG00000072720	Myo18b	-Inf	0.021651
ENSMUSG00000072852	2310040G07Rik	-Inf	0.042407
ENSMUSG00000073380	Arrdc5	-Inf	0.036563
ENSMUSG00000073680	Tmem88b	-Inf	0.034615
ENSMUSG00000074158	9830147E19Rik	-Inf	0.035466
ENSMUSG00000074480	Mex3a	-Inf	0.046478
ENSMUSG00000074867	Zfp808	-8.35968	0.0303
ENSMUSG00000075014	Gm10800	-2.29268	0.049507
ENSMUSG00000075415	Fnbp1	-1.69909	0.043261
ENSMUSG00000078157	4931440F15Rik	-5.66142	0.016343
ENSMUSG00000078716	Tmem8b	-2.63749	0.035294
ENSMUSG00000078815	Cacng6	-Inf	0.01732
ENSMUSG00000079553	Kifc1	-Inf	0.021706
ENSMUSG00000079620	Muc4	-Inf	0.034166
ENSMUSG00000079654	Prrt4	-Inf	0.044065
ENSMUSG00000084884	Gm12289	-Inf	0.045057
ENSMUSG00000084890	A830036E02Rik	-1.53363	0.024695
ENSMUSG00000085039	Gm15927	-Inf	0.026393
ENSMUSG00000085054	Gm15834	-Inf	0.030186
ENSMUSG00000085623	Gm16041	-Inf	0.012424
ENSMUSG00000086602	Gm15609	-Inf	0.032722
ENSMUSG00000086837	Gm16618	-6.79873	0.032425
ENSMUSG00000087064	Sap30bpos	-Inf	0.025561
ENSMUSG00000087340	Gm15228	-Inf	0.039515
ENSMUSG00000087354	4930404I05Rik	-Inf	0.049658
ENSMUSG00000087579	1500017E21Rik	-Inf	0.048089
ENSMUSG00000090115	Usp49	-3.26234	0.048645
ENSMUSG00000091455	Otogl	-Inf	0.046097

ENSMUSG00000092323	BB365896	-Inf	0.022499
ENSMUSG00000092486	2610524H06Rik	-6.08227	0.013316
ENSMUSG00000093502	Gm20700	-5.94029	0.045612
ENSMUSG00000093598	A730085K08Rik	-Inf	0.046045
ENSMUSG00000096916	Zfp850	-Inf	0.037861
ENSMUSG00000097335	Gm26563	-6.90729	0.040191
ENSMUSG00000097380	Gm26816	-4.45331	0.04541
ENSMUSG00000097476	Gm26583	-Inf	0.034214
ENSMUSG00000097479	Gm26582	-3.53473	0.046167
ENSMUSG00000100975	Gm28875	-Inf	0.049017
ENSMUSG00000102139	Gm37109	-Inf	0.043454
ENSMUSG00000102206	Pcdha11	-Inf	0.002291
ENSMUSG00000102440	Pcdhga9	-2.84525	0.04706
ENSMUSG00000102481	Gm37925	-Inf	0.01222
ENSMUSG00000102854	C130023A14Rik	-6.01771	0.033944
ENSMUSG00000102919	Gm37726	-Inf	0.031181
ENSMUSG00000103101	Gm37048	-Inf	0.048351
ENSMUSG00000103138	Gm2238	-8.76921	0.016168
ENSMUSG00000103348	Gm37053	-Inf	0.0164
ENSMUSG00000103377	Gm37180	-Inf	0.013801
ENSMUSG00000103630	Gm37242	-Inf	0.032004
ENSMUSG00000103734	Gm37651	-Inf	0.039381
ENSMUSG00000103748	Gm38243	-5.13717	0.020759
ENSMUSG00000103770	Pcdha9	-Inf	0.027777
ENSMUSG00000104044	Gm37566	-Inf	0.040693
ENSMUSG00000104154	Gm38104	-6.54922	0.036855
ENSMUSG00000105083	Gm42699	-6.79401	0.044658
ENSMUSG00000105229	Gm43149	-Inf	0.023812
ENSMUSG00000105528	Gm43519	-Inf	0.014086
ENSMUSG00000105622	Gm42615	-Inf	0.011727
ENSMUSG00000105707	Gm43753	-8.40514	0.017575
ENSMUSG00000105776	Gm43292	-6.93552	0.013415

ENSMUSG00000105936	Gm43544	-Inf	0.024296
ENSMUSG00000106048	Gm42444	-5.77585	0.039502
ENSMUSG00000106099	Gm42664	-Inf	0.033793
ENSMUSG00000106408	Gm43321	-Inf	0.01927
ENSMUSG00000106539	Gm43670	-1.33448	0.044183
ENSMUSG00000106696	Gm42729	-Inf	0.006434
ENSMUSG00000106717	Gm42798	-8.43989	0.006363
ENSMUSG00000107045	Gm43636	-Inf	0.024994
ENSMUSG00000107083	Gm43313	-5.83051	0.033877
ENSMUSG00000107362	Gm42724	-2.52944	0.018892
ENSMUSG00000107707	RP23-140M6.1	-6.39859	0.015511
ENSMUSG00000107884	RP23-334M3.6	-Inf	0.031728
ENSMUSG00000108053	RP24-317F6.7	-Inf	0.037895
ENSMUSG00000108076	RP23-296N5.4	-5.78432	0.018022
ENSMUSG00000108353	RP24-144C5.1	-Inf	0.016292
ENSMUSG00000108522	RP23-385F8.2	-4.95969	0.046039

80

Supplementary Table 4. The list of genes (145) that are upregulated in $Glu^{S1L2/3}$ neurons of CCI maternal mice relative to those of sham controls (fold change ≥ 2, $P < 0.05$).

Ensembl Gene ID	Gene Symbol	Log2 Fold Change CCI over sham	P value (CCI over sham)
ENSMUSG00000003657	Calb2	+Inf	0.000136
ENSMUSG00000027399	Il1a	4.582635	0.000441
ENSMUSG00000013523	Bcas1	+Inf	0.000652
ENSMUSG00000001768	Rin2	8.712169	0.000725
ENSMUSG00000073062	Zxdb	5.815225	0.000731
ENSMUSG00000002699	Lcp2	9.593719	0.001223
ENSMUSG00000020120	Plek	3.984564	0.001592
ENSMUSG00000029819	Npy	+Inf	0.001606
ENSMUSG00000074622	Mafb	6.706809	0.001634
ENSMUSG00000054675	Tmem119	4.255723	0.002146
ENSMUSG00000035151	Elmod2	8.642843	0.002412
ENSMUSG00000068798	Rap1a	8.369298	0.002527
ENSMUSG00000031504	Rab20	8.321734	0.003159
ENSMUSG00000060636	Rpl35a	7.696941	0.003386
ENSMUSG00000021238	Aldh6a1	5.287063	0.003633
ENSMUSG00000093677	Gm20712	+Inf	0.003798
ENSMUSG00000069662	Marcks	3.203564	0.003834
ENSMUSG00000042350	Arel1	2.946682	0.004935
ENSMUSG00000018930	Ccl4	2.91923	0.005555
ENSMUSG00000044231	Nhlrc1	+Inf	0.005625
ENSMUSG00000087651	1500009L16Rik	+Inf	0.005677
ENSMUSG00000022500	Litaf	5.341914	0.005836
ENSMUSG00000015396	Cd83	2.949974	0.006351
ENSMUSG00000048988	Elfn1	+Inf	0.006842
ENSMUSG00000024621	Csf1r	3.611479	0.006877
ENSMUSG00000070880	Gad1	4.137637	0.007235
ENSMUSG00000075528	Aarsd1	6.024336	0.007739
ENSMUSG00000003283	Hck	+Inf	0.007767

ENSMUSG00000021665	Hexb	2.740991	0.008331
ENSMUSG00000039450	Dcxr	+Inf	0.008378
ENSMUSG00000018593	Sparc	2.848511	0.008551
ENSMUSG00000044770	Scml4	+Inf	0.008757
ENSMUSG00000025537	Phkg1	+Inf	0.009216
ENSMUSG00000018774	Cd68	3.762471	0.009634
ENSMUSG00000047832	Cdca4	+Inf	0.009798
ENSMUSG00000042505	Sdhaf3	6.508964	0.010405
ENSMUSG00000053519	Kcnip1	+Inf	0.01046
ENSMUSG00000025666	Tmem47	2.598054	0.010472
ENSMUSG00000095432	Zfp748	7.712307	0.010683
ENSMUSG00000036206	Sh3bp4	8.220328	0.010953
ENSMUSG00000032410	Xrn1	3.01732	0.011538
ENSMUSG00000046447	Camk2n1	2.199598	0.012344
ENSMUSG00000058748	Zfp958	+Inf	0.012691
ENSMUSG00000043008	Klhl6	+Inf	0.012896
ENSMUSG00000034652	Cd300a	8.096425	0.013524
ENSMUSG00000073411	H2-D1	2.739938	0.013785
ENSMUSG00000036968	Cnpy4	+Inf	0.013926
ENSMUSG00000021189	Atxn3	4.097297	0.014223
ENSMUSG00000047735	Samd9l	7.243811	0.014551
ENSMUSG00000004837	Grap	7.348709	0.015023
ENSMUSG00000085715	Tsix	2.977303	0.015036
ENSMUSG00000024048	Myl12a	3.528449	0.016198
ENSMUSG00000020334	Slc22a4	8.17565	0.016657
ENSMUSG00000000982	Ccl3	2.923801	0.017283
ENSMUSG00000037405	Icam1	5.337148	0.017286
ENSMUSG00000070601	Vmn2r84	+Inf	0.017417
ENSMUSG00000079235	Ccdc13	+Inf	0.017577
ENSMUSG00000021613	Hapln1	+Inf	0.0176
ENSMUSG00000036887	C1qa	2.399206	0.018012
ENSMUSG00000041515	Irf8	+Inf	0.018663

ENSMUSG00000030605	Mfge8	7.879882	0.018841
ENSMUSG00000021532	Fastkd3	+Inf	0.018842
ENSMUSG00000004609	Cd33	6.042104	0.019007
ENSMUSG00000023942	Slc29a1	+Inf	0.019071
ENSMUSG00000029119	Man2b2	+Inf	0.019114
ENSMUSG00000062210	Tnfaip8	+Inf	0.019207
ENSMUSG00000007613	Tgfbr1	4.410902	0.019676
ENSMUSG00000047123	Ticam1	+Inf	0.02021
ENSMUSG00000079242	C730034F03Rik	9.333748	0.021196
ENSMUSG00000075284	Wipf1	4.415762	0.021815
ENSMUSG00000017057	Il13ra1	5.621724	0.022175
ENSMUSG00000002147	Stat6	+Inf	0.022286
ENSMUSG00000009214	Tmem8c	+Inf	0.022538
ENSMUSG00000044367	Slc16a13	+Inf	0.022637
ENSMUSG00000031639	Tlr3	+Inf	0.023035
ENSMUSG00000032854	Ugt8a	6.018669	0.023504
ENSMUSG00000046034	Otulin	+Inf	0.024011
ENSMUSG00000032667	Pon2	4.141731	0.02405
ENSMUSG00000036905	C1qb	2.192438	0.024298
ENSMUSG00000030083	Abtb1	2.79424	0.024916
ENSMUSG00000051067	Lingo3	3.581425	0.02534
ENSMUSG00000086407	Gm14123	+Inf	0.025948
ENSMUSG00000037653	Kctd8	7.807895	0.026746
ENSMUSG00000020399	Havcr2	+Inf	0.027033
ENSMUSG00000034343	Ube2f	2.521367	0.027125
ENSMUSG00000062169	Cnih4	3.769263	0.027187
ENSMUSG00000061740	Cyp2d22	+Inf	0.027919
ENSMUSG00000025017	Pik3ap1	6.786637	0.030233
ENSMUSG00000020092	Pald1	+Inf	0.030366
ENSMUSG00000048965	Mrgpre	+Inf	0.030724
ENSMUSG00000024816	Frmd8	6.576336	0.030789
ENSMUSG00000026787	Gad2	4.196537	0.03136

ENSMUSG00000085498	Gm14023	+Inf	0.032265
ENSMUSG00000036863	Syde2	+Inf	0.033084
ENSMUSG00000034595	Ppp1r18	4.752886	0.033236
ENSMUSG00000057335	Cep170	2.216243	0.033491
ENSMUSG00000068551	Zfp467	6.974691	0.033648
ENSMUSG00000091387	Gcnt4	+Inf	0.033932
ENSMUSG00000049436	Upk1b	9.200594	0.034572
ENSMUSG00000026841	Fibcd1	+Inf	0.034608
ENSMUSG00000036885	Arhgef26	3.15776	0.035421
ENSMUSG00000022901	Cd86	6.526797	0.035707
ENSMUSG00000090164	BC035044	6.977093	0.035888
ENSMUSG00000048163	Selplg	2.543418	0.035976
ENSMUSG00000049791	Fzd4	2.88739	0.036237
ENSMUSG00000036983	Tfb1m	+Inf	0.036316
ENSMUSG00000019936	Epyc	+Inf	0.036532
ENSMUSG00000105293	Gm42843	2.797529	0.037306
ENSMUSG00000049775	Tmsb4x	1.862995	0.037636
ENSMUSG00000027239	Mdk	+Inf	0.038247
ENSMUSG00000031907	Zfp90	+Inf	0.038625
ENSMUSG00000026749	Nek6	2.790804	0.038632
ENSMUSG00000066568	Lsm14a	2.974808	0.038745
ENSMUSG00000036402	Gng12	3.099899	0.039519
ENSMUSG00000040446	Rprd1a	2.910607	0.039688
ENSMUSG00000040253	Gbp7	+Inf	0.039808
ENSMUSG00000048895	Cdk5r1	2.211606	0.040429
ENSMUSG00000067377	Tspan6	6.408649	0.04049
ENSMUSG00000029580	Actb	1.847673	0.040515
ENSMUSG00000022009	Nufip1	3.5993	0.040658
ENSMUSG00000028631	Kcnq4	+Inf	0.041004
ENSMUSG00000004558	Ndrg2	2.084282	0.041182
ENSMUSG00000030342	Cd9	4.167293	0.041511
ENSMUSG00000024507	Hsd17b4	3.46918	0.041672

ENSMUSG00000036555	Iqce	2.554187	0.041978
ENSMUSG00000091955	Gm9844	2.591223	0.042578
ENSMUSG00000047067	Dusp28	4.669155	0.0427
ENSMUSG00000020037	Rfx4	+Inf	0.043064
ENSMUSG00000007659	Bcl2l1	2.173262	0.043103
ENSMUSG00000034919	Ttc22	+Inf	0.04312
ENSMUSG00000041911	Dlx1	+Inf	0.045025
ENSMUSG00000090546	Cdr1	1.812925	0.045195
ENSMUSG00000030446	Zfp273	4.88849	0.045555
ENSMUSG00000018008	Cyth4	+Inf	0.045778
ENSMUSG00000032425	Zfp949	3.560635	0.045937
ENSMUSG00000037669	Ldah	2.945301	0.04624
ENSMUSG00000021508	Cxcl14	3.286737	0.046539
ENSMUSG00000022817	Itgb5	6.778376	0.047033
ENSMUSG00000057396	Zfp759	+Inf	0.047428
ENSMUSG00000031197	Vbp1	2.219951	0.047463
ENSMUSG00000020610	Amz2	5.787355	0.047869
ENSMUSG00000035696	Rnf38	3.510875	0.047979
ENSMUSG00000104273	A530064N14Rik	+Inf	0.048469
ENSMUSG00000054409	Tmem74	+Inf	0.049495
ENSMUSG00000036880	Acaa2	+Inf	0.049912

Supplementary Table 5. The list of genes (426) that are downregulated in Glu$^{S1L2/3}$ neurons of CCI maternal mice relative to those of sham controls (fold change ≤ 0.5, P < 0.05).

Ensembl Gene ID	Gene Symbol	Log2 Fold Change CCI over sham	P value (CCI over sham)
ENSMUSG00000048920	Fkrp	-Inf	2.38E-05
ENSMUSG00000044847	Lsm11	-Inf	3.33E-05
ENSMUSG00000027207	Galk2	-Inf	5.60E-05
ENSMUSG00000037905	Bri3bp	-Inf	9.55E-05
ENSMUSG00000103432	6720464F23Rik	-10.3995	0.000114
ENSMUSG00000079555	Haus3	-Inf	0.000117
ENSMUSG00000037339	Fam53a	-Inf	0.000142
ENSMUSG00000052305	Hbb-bs	-7.84905	0.000172
ENSMUSG00000048537	Phldb1	-Inf	0.000177
ENSMUSG00000026224	4933407L21Rik	-Inf	0.000181
ENSMUSG00000061028	Clasrp	-3.78355	0.000216
ENSMUSG00000089872	Rps6kc1	-4.16564	0.000276
ENSMUSG00000064210	Ano6	-4.10402	0.000298
ENSMUSG00000024142	Mlst8	-Inf	0.000302
ENSMUSG00000020387	Jade2	-7.27335	0.000307
ENSMUSG00000078190	Dnm3os	-10.0027	0.000329
ENSMUSG00000069917	Hba-a2	-6.74336	0.000387
ENSMUSG00000050605	Zfp61	-Inf	0.000419
ENSMUSG00000013419	Zfp651	-Inf	0.000426
ENSMUSG00000039813	Tbc1d2	-9.80999	0.000459
ENSMUSG00000075014	Gm10800	-4.35934	0.000493
ENSMUSG00000027014	Cwc22	-Inf	0.000509
ENSMUSG00000016200	Syt14	-7.56548	0.000676
ENSMUSG00000103472	Pcdhga7	-Inf	0.000678
ENSMUSG00000026856	Dolpp1	-Inf	0.000742
ENSMUSG00000037366	Pafah2	-Inf	0.000821
ENSMUSG00000035919	Bbs9	-8.77037	0.000848
ENSMUSG00000058454	Dhcr7	-7.80479	0.000896

ENSMUSG00000032609	Klhdc8b	-4.38927	0.000939
ENSMUSG00000024172	St6gal2	-4.46615	0.00097
ENSMUSG00000024170	Telo2	-Inf	0.000984
ENSMUSG00000020656	Grhl1	-6.1339	0.000992
ENSMUSG00000061762	Tac1	-Inf	0.001011
ENSMUSG00000071103	1700029J07Rik	-Inf	0.001014
ENSMUSG00000106212	Gm43112	-9.34034	0.00104
ENSMUSG00000063895	Nupl1	-7.13589	0.001257
ENSMUSG00000038524	Fchsd1	-Inf	0.001314
ENSMUSG00000075590	Nrbp2	-4.95041	0.001325
ENSMUSG00000023923	Tbc1d5	-Inf	0.001331
ENSMUSG00000021962	Dcp1a	-6.13363	0.001341
ENSMUSG00000069919	Hba-a1	-5.98563	0.00146
ENSMUSG00000045466	Zfp956	-7.82662	0.001761
ENSMUSG00000036918	Ttc7	-Inf	0.001899
ENSMUSG00000028948	Nol9	-Inf	0.001957
ENSMUSG00000070002	Ell	-5.9382	0.001961
ENSMUSG00000025962	Fastkd2	-Inf	0.002006
ENSMUSG00000033083	Tbc1d4	-Inf	0.002093
ENSMUSG00000045252	Zfp574	-6.19953	0.002187
ENSMUSG00000022237	Ankrd33b	-2.62333	0.002219
ENSMUSG00000046562	Unc119b	-Inf	0.002254
ENSMUSG00000036686	Cc2d1a	-Inf	0.00228
ENSMUSG00000046947	Adck2	-Inf	0.00238
ENSMUSG00000051412	Vamp7	-7.4722	0.002518
ENSMUSG00000024642	Tle4	-6.63342	0.002652
ENSMUSG00000042659	Arrdc4	-Inf	0.002722
ENSMUSG00000049323	Smcr8	-8.62895	0.002731
ENSMUSG00000038259	Gdf5	-Inf	0.00304
ENSMUSG00000046185	Zfp84	-Inf	0.003092
ENSMUSG00000030386	Zfp606	-Inf	0.003097
ENSMUSG00000022096	Hr	-Inf	0.003264

ENSMUSG00000034898	Filip1	-Inf	0.003335
ENSMUSG00000026135	Zfp142	-3.63803	0.003403
ENSMUSG00000001998	Ap4e1	-Inf	0.003416
ENSMUSG00000027584	Oprl1	-Inf	0.00348
ENSMUSG00000036167	Pphln1	-9.44678	0.003601
ENSMUSG00000038260	Trpm4	-Inf	0.003643
ENSMUSG00000007877	Tcap	-Inf	0.004092
ENSMUSG00000031834	Pik3r2	-2.65242	0.004125
ENSMUSG00000040356	Skiv2l	-3.63903	0.004137
ENSMUSG00000030681	Mvp	-Inf	0.004304
ENSMUSG00000059839	Zfp874b	-Inf	0.00435
ENSMUSG00000102317	Gm37628	-Inf	0.004643
ENSMUSG00000035575	Utp6	-Inf	0.004721
ENSMUSG00000020674	Pxdn	-4.52845	0.004779
ENSMUSG00000018809	Smyd4	-Inf	0.00492
ENSMUSG00000052613	Pcdh15	-4.84711	0.005075
ENSMUSG00000102758	Naaladl2	-Inf	0.005104
ENSMUSG00000004054	Map3k11	-Inf	0.00513
ENSMUSG00000034165	Ccnd3	-7.87889	0.005315
ENSMUSG00000040420	Cdh18	-Inf	0.00556
ENSMUSG00000104671	Gm43062	-Inf	0.005619
ENSMUSG00000027400	Pdyn	-7.79062	0.005778
ENSMUSG00000025737	Wdr24	-Inf	0.00583
ENSMUSG00000048310	Pskh1	-Inf	0.005865
ENSMUSG00000027022	Xirp2	-Inf	0.005866
ENSMUSG00000032519	Slc25a38	-Inf	0.005877
ENSMUSG00000045875	Adra1a	-Inf	0.006142
ENSMUSG00000108522	RP23-385F8.2	-Inf	0.006193
ENSMUSG00000073940	Hbb-bt	-Inf	0.006243
ENSMUSG00000091028	Gm10722	-5.23862	0.006326
ENSMUSG00000036502	Tmem255a	-Inf	0.006577
ENSMUSG00000042216	Sgsm1	-3.02067	0.006584

ENSMUSG00000028007	Snx7	-8.69478	0.006683
ENSMUSG00000099931	Gm29358	-Inf	0.006766
ENSMUSG00000070426	Rnf121	-Inf	0.006773
ENSMUSG00000061979	Wbscr16	-Inf	0.006894
ENSMUSG00000104655	Gm43317	-Inf	0.006895
ENSMUSG00000031540	Kat6a	-4.68792	0.006902
ENSMUSG00000035258	Abi3bp	-Inf	0.007083
ENSMUSG00000029442	Wdr66	-8.81377	0.007091
ENSMUSG00000058153	Sez6l	-2.58316	0.007204
ENSMUSG00000081137	BC022960	-Inf	0.007259
ENSMUSG00000040359	Ufl1	-6.18452	0.007308
ENSMUSG00000096146	Kcnj11	-Inf	0.007322
ENSMUSG00000015290	Ubl4a	-3.64571	0.007347
ENSMUSG00000031134	Rbmx	-6.45921	0.007452
ENSMUSG00000039552	Rsph4a	-Inf	0.007522
ENSMUSG00000108621	Gm37494	-Inf	0.007828
ENSMUSG00000026816	Gtf3c5	-4.42587	0.008346
ENSMUSG00000006342	Susd2	-Inf	0.008514
ENSMUSG00000105776	Gm43292	-Inf	0.008779
ENSMUSG00000038895	Zfp653	-5.87981	0.008925
ENSMUSG00000025395	Prim1	-Inf	0.008951
ENSMUSG00000026188	Tmem169	-Inf	0.009122
ENSMUSG00000032064	Dixdc1	-3.0872	0.009198
ENSMUSG00000032263	Bckdhb	-Inf	0.009221
ENSMUSG00000038838	Vars2	-Inf	0.009262
ENSMUSG00000106265	Gm42490	-Inf	0.009375
ENSMUSG00000075553	Gm5464	-Inf	0.009561
ENSMUSG00000045201	Lrrc3b	-Inf	0.00963
ENSMUSG00000030823	9130019O22Rik	-Inf	0.009651
ENSMUSG00000023938	Aars2	-8.77287	0.009673
ENSMUSG00000097546	Gm26749	-Inf	0.009682
ENSMUSG00000104156	Gm38102	-Inf	0.009799

ENSMUSG00000029416	Slc15a4	-Inf	0.009838
ENSMUSG00000001506	Col1a1	-Inf	0.010026
ENSMUSG00000075224	Lrrc55	-6.07011	0.010255
ENSMUSG00000019857	Asf1a	-Inf	0.010496
ENSMUSG00000102861	Gm37637	-Inf	0.010502
ENSMUSG00000059921	Unc5c	-6.51222	0.010576
ENSMUSG00000087479	Gm16835	-7.03349	0.010581
ENSMUSG00000044345	Marveld1	-Inf	0.010585
ENSMUSG00000075415	Fnbp1	-2.91728	0.010763
ENSMUSG00000019647	Sema6a	-7.2537	0.010791
ENSMUSG00000026826	Nr4a2	-4.22685	0.010867
ENSMUSG00000047793	Sned1	-Inf	0.01089
ENSMUSG00000108081	RP23-415I19.4	-Inf	0.010916
ENSMUSG00000102854	C130023A14Rik	-Inf	0.010937
ENSMUSG00000025665	Rps6ka6	-Inf	0.010981
ENSMUSG00000000126	Wnt9a	-Inf	0.011092
ENSMUSG00000074037	Mc1r	-8.61889	0.011592
ENSMUSG00000006641	Slc5a6	-Inf	0.011883
ENSMUSG00000045441	Gprin3	-6.99805	0.011935
ENSMUSG00000040296	Ddx58	-Inf	0.012173
ENSMUSG00000032558	Nphp3	-9.11943	0.01229
ENSMUSG00000089739	Gm20431	-Inf	0.012408
ENSMUSG00000068580	Zfyve19	-Inf	0.01244
ENSMUSG00000026469	Xpr1	-3.7659	0.012448
ENSMUSG00000001280	Sp1	-Inf	0.012477
ENSMUSG00000047021	Ccdc108	-Inf	0.012688
ENSMUSG00000020846	Fam101b	-Inf	0.012941
ENSMUSG00000085133	B930095G15Rik	-3.11784	0.012986
ENSMUSG00000024982	Zdhhc6	-5.98785	0.013161
ENSMUSG00000040690	Col16a1	-Inf	0.013289
ENSMUSG00000022895	Ets2	-2.88277	0.013803
ENSMUSG00000047415	Gpr68	-Inf	0.014338

ENSMUSG00000105804	Gm43654	-Inf	0.014538
ENSMUSG00000041303	Gtf3c3	-4.70475	0.014572
ENSMUSG00000002279	Lmf1	-Inf	0.014619
ENSMUSG00000020661	Dnmt3a	-2.14885	0.014654
ENSMUSG00000039981	Zc3h12d	-Inf	0.015129
ENSMUSG00000010045	Tmem115	-5.27268	0.015286
ENSMUSG00000005873	Reep5	-1.96725	0.015326
ENSMUSG00000102516	Gm38340	-Inf	0.01533
ENSMUSG00000026404	Ddx59	-Inf	0.015342
ENSMUSG00000036295	Lrrn3	-4.67164	0.015538
ENSMUSG00000102543	Pcdhgc5	-4.09188	0.015687
ENSMUSG00000020212	Mdm1	-Inf	0.015709
ENSMUSG00000004929	Thop1	-3.87284	0.01574
ENSMUSG00000104822	Gm42967	-Inf	0.015923
ENSMUSG00000028661	Epha8	-Inf	0.016052
ENSMUSG00000053094	Tmem248	-3.35307	0.016117
ENSMUSG00000032396	Dis3l	-6.63565	0.016137
ENSMUSG00000034156	Bzrap1	-1.9465	0.016177
ENSMUSG00000034551	Hdx	-Inf	0.016304
ENSMUSG00000053334	Ficd	-7.08109	0.016315
ENSMUSG00000042745	Id1	-Inf	0.016552
ENSMUSG00000027075	Slc43a1	-Inf	0.016826
ENSMUSG00000039007	Cpq	-Inf	0.017368
ENSMUSG00000104257	Gm20172	-Inf	0.01761
ENSMUSG00000044072	Eml6	-3.9281	0.017636
ENSMUSG00000038214	Bend3	-Inf	0.017637
ENSMUSG00000031730	Dhodh	-Inf	0.017763
ENSMUSG00000032017	Grik4	-4.41006	0.018111
ENSMUSG00000102336	Gm37233	-Inf	0.01814
ENSMUSG00000026227	2810459M11Rik	-Inf	0.018518
ENSMUSG00000040105	Ppapdc2	-Inf	0.018606
ENSMUSG00000092375	A730060N03Rik	-Inf	0.01921

ENSMUSG00000103138	Gm2238	-Inf	0.019258
ENSMUSG00000103272	Gm37914	-Inf	0.019332
ENSMUSG00000028195	Cyr61	-3.58658	0.019392
ENSMUSG00000018634	Crhr1	-4.63189	0.019737
ENSMUSG00000047824	Pygo2	-Inf	0.01974
ENSMUSG00000039990	Edrf1	-2.72925	0.020031
ENSMUSG00000016526	Dyrk3	-6.25065	0.020231
ENSMUSG00000029221	Slc30a9	-2.39475	0.020273
ENSMUSG00000103375	Gm37181	-7.86293	0.020313
ENSMUSG00000053581	Zfand2a	-Inf	0.020434
ENSMUSG00000027298	Tyro3	-2.07706	0.020568
ENSMUSG00000047037	Nipa1	-Inf	0.020626
ENSMUSG00000072568	Fam84b	-Inf	0.020684
ENSMUSG00000107576	RP23-236J3.4	-7.17871	0.020787
ENSMUSG00000000538	Tom1l2	-2.97258	0.021096
ENSMUSG00000069114	Zbtb10	-4.46125	0.02116
ENSMUSG00000033458	Fan1	-6.62301	0.021267
ENSMUSG00000034930	Rtkn	-5.2623	0.021338
ENSMUSG00000093436	Gm20646	-Inf	0.021387
ENSMUSG00000027999	Pla2g12a	-5.06459	0.021761
ENSMUSG00000075470	Alg10b	-Inf	0.022006
ENSMUSG00000033365	Ipo13	-3.21081	0.022171
ENSMUSG00000036834	Plch1	-5.68238	0.022201
ENSMUSG00000097274	Gm26564	-Inf	0.022202
ENSMUSG00000027175	Tcp11l1	-Inf	0.022514
ENSMUSG00000043587	Pxylp1	-Inf	0.022562
ENSMUSG00000038268	Ovca2	-4.62856	0.022983
ENSMUSG00000029648	Flt1	-Inf	0.023008
ENSMUSG00000031483	Erlin2	-Inf	0.023009
ENSMUSG00000045896	Paip2b	-5.61834	0.023202
ENSMUSG00000027164	Traf6	-3.8471	0.023224
ENSMUSG00000052296	Ppp6r1	-4.52807	0.023456

ENSMUSG00000022894	Adamts5	-Inf	0.023457
ENSMUSG00000035431	Sstr1	-Inf	0.023467
ENSMUSG00000039021	Ttc16	-Inf	0.02348
ENSMUSG00000038212	Hiatl1	-4.28945	0.023523
ENSMUSG00000033416	Gucd1	-6.00804	0.023747
ENSMUSG00000039116	Adgrg6	-Inf	0.023829
ENSMUSG00000022967	Ifnar1	-3.26424	0.023876
ENSMUSG00000049680	Urgcp	-4.37171	0.023958
ENSMUSG00000078429	Ctdsp2	-2.66314	0.02396
ENSMUSG00000050963	Kcns2	-Inf	0.024082
ENSMUSG00000075015	Gm10801	-3.86922	0.024119
ENSMUSG00000035486	Plk5	-2.72853	0.024525
ENSMUSG00000104641	Gm43290	-Inf	0.024553
ENSMUSG00000035769	Xylb	-Inf	0.024578
ENSMUSG00000058883	Zfp708	-Inf	0.024714
ENSMUSG00000069206	Zfp874a	-6.86782	0.024843
ENSMUSG00000033629	Hacd3	-2.00045	0.024933
ENSMUSG00000058975	Kcnc1	-2.30324	0.024979
ENSMUSG00000085623	Gm16041	-Inf	0.025029
ENSMUSG00000044254	Pcsk9	-Inf	0.025092
ENSMUSG00000022100	Xpo7	-3.22226	0.02515
ENSMUSG00000029217	Tec	-Inf	0.025372
ENSMUSG00000097121	D130020L05Rik	-Inf	0.0254
ENSMUSG00000026479	Lamc2	-Inf	0.025479
ENSMUSG00000097312	Gm26870	-4.46373	0.025512
ENSMUSG00000018974	Sart3	-3.00655	0.025653
ENSMUSG00000026198	Abcb6	-4.63519	0.025743
ENSMUSG00000054057	A930004D18Rik	-Inf	0.02586
ENSMUSG00000027300	Ubox5	-8.63539	0.025891
ENSMUSG00000024357	Sil1	-Inf	0.026084
ENSMUSG00000014355	Anapc1	-2.29838	0.026285
ENSMUSG00000087095	Emx2os	-Inf	0.0263

ENSMUSG00000078815	Cacng6	-Inf	0.026314
ENSMUSG00000026241	Nppc	-Inf	0.026405
ENSMUSG00000053134	Supt7l	-Inf	0.026648
ENSMUSG00000032560	Dnajc13	-2.45478	0.026666
ENSMUSG00000042804	Gpr153	-Inf	0.02673
ENSMUSG00000029716	Tfr2	-8.25785	0.02674
ENSMUSG00000036879	Phkb	-4.19947	0.026749
ENSMUSG00000027184	Caprin1	-2.43577	0.02683
ENSMUSG00000025348	Itga7	-Inf	0.027018
ENSMUSG00000102874	Gm37137	-8.78509	0.027024
ENSMUSG00000032119	Hinfp	-Inf	0.027067
ENSMUSG00000038564	Ift172	-2.77201	0.027237
ENSMUSG00000042500	Ago4	-Inf	0.027268
ENSMUSG00000020413	Hus1	-6.86944	0.027307
ENSMUSG00000106491	Gm42446	-Inf	0.027809
ENSMUSG00000102401	Gm37864	-Inf	0.027824
ENSMUSG00000032718	Mansc1	-Inf	0.027843
ENSMUSG00000028854	Slc9a1	-4.77044	0.028087
ENSMUSG00000031239	Itm2a	-Inf	0.028118
ENSMUSG00000021758	Ddx4	-Inf	0.02831
ENSMUSG00000002897	Il17ra	-5.0261	0.028564
ENSMUSG00000042229	Rabif	-Inf	0.028678
ENSMUSG00000031906	Smpd3	-5.0028	0.028732
ENSMUSG00000082315	Gm16523	-4.93184	0.028776
ENSMUSG00000003123	Lipe	-Inf	0.029026
ENSMUSG00000108353	RP24-144C5.1	-Inf	0.029128
ENSMUSG00000034473	Sec22a	-5.28343	0.029291
ENSMUSG00000020546	Stxbp4	-Inf	0.029663
ENSMUSG00000079429	Mroh2a	-Inf	0.029745
ENSMUSG00000027843	Ptpn22	-Inf	0.029911
ENSMUSG00000028782	Adgrb2	-2.64846	0.030044
ENSMUSG00000039308	Ndst2	-Inf	0.03013

ENSMUSG00000032816	Igdcc4	-6.69289	0.030134
ENSMUSG00000103348	Gm37053	-Inf	0.030168
ENSMUSG00000107331	Gm42732	-Inf	0.030242
ENSMUSG00000006705	Pknox1	-5.12767	0.030308
ENSMUSG00000095280	Gm21738	-4.29383	0.030427
ENSMUSG00000002984	Tomm40	-2.47465	0.030453
ENSMUSG00000040097	Flywch1	-1.99316	0.030501
ENSMUSG00000038205	Prkab2	-2.45392	0.030645
ENSMUSG00000044916	1700029I15Rik	-Inf	0.03067
ENSMUSG00000029263	Pigg	-Inf	0.03071
ENSMUSG00000058093	Zfp729b	-Inf	0.030783
ENSMUSG00000048485	Zbtb8b	-Inf	0.030841
ENSMUSG00000043857	Mgat5b	-5.22802	0.030887
ENSMUSG00000054381	Zfp747	-Inf	0.030887
ENSMUSG00000025745	Hadha	-4.95768	0.031125
ENSMUSG00000078762	Haus5	-Inf	0.031741
ENSMUSG00000045106	Ccdc73	-Inf	0.031828
ENSMUSG00000050711	Scg2	-1.78263	0.032312
ENSMUSG00000028423	Nfx1	-1.85215	0.03252
ENSMUSG00000104061	Gm37953	-8.18673	0.032742
ENSMUSG00000079553	Kifc1	-Inf	0.03295
ENSMUSG00000074867	Zfp808	-Inf	0.033084
ENSMUSG00000072720	Myo18b	-Inf	0.03325
ENSMUSG00000103565	Sgsm3	-Inf	0.033434
ENSMUSG00000103046	Gm37309	-Inf	0.033712
ENSMUSG00000028518	Prkaa2	-2.04307	0.033727
ENSMUSG00000017167	Cntnap1	-3.19195	0.03383
ENSMUSG00000052221	Ppp1r36	-Inf	0.03404
ENSMUSG00000010097	Nxf1	-1.72745	0.0341
ENSMUSG00000057788	Ddx49	-5.4196	0.034787
ENSMUSG00000102602	A930004J17Rik	-Inf	0.03496
ENSMUSG00000105083	Gm42699	-Inf	0.035058

ENSMUSG00000021702	Thbs4	-Inf	0.035094
ENSMUSG00000024579	Pcyox1l	-Inf	0.035143
ENSMUSG00000104435	Gm37422	-Inf	0.03527
ENSMUSG00000020474	Polm	-Inf	0.035278
ENSMUSG00000074629	4930518I15Rik	-Inf	0.035405
ENSMUSG00000046139	Patl1	-3.01245	0.035424
ENSMUSG00000029714	Gigyf1	-2.58717	0.035595
ENSMUSG00000094840	Muc3a	-5.76344	0.035658
ENSMUSG00000033740	St18	-Inf	0.035708
ENSMUSG00000028980	H6pd	-Inf	0.035715
ENSMUSG00000028927	Padi2	-Inf	0.035943
ENSMUSG00000023439	Gnb3	-Inf	0.036077
ENSMUSG00000005225	Plekha8	-Inf	0.03639
ENSMUSG00000090100	Ttbk2	-2.41201	0.036555
ENSMUSG00000040152	Thbs1	-Inf	0.036633
ENSMUSG00000016918	Sulf1	-Inf	0.036741
ENSMUSG00000030536	Iqgap1	-1.99766	0.036902
ENSMUSG00000075419	Dolk	-5.7428	0.036904
ENSMUSG00000102579	Gm37965	-Inf	0.037069
ENSMUSG00000070425	Xntrpc	-Inf	0.037236
ENSMUSG00000103482	Gm37999	-Inf	0.037259
ENSMUSG00000031066	Usp11	-1.79312	0.037698
ENSMUSG00000108572	RP24-243C16.2	-Inf	0.037974
ENSMUSG00000014782	Plekhg4	-Inf	0.038012
ENSMUSG00000025512	Chid1	-3.77953	0.038056
ENSMUSG00000049796	Crh	-Inf	0.038198
ENSMUSG00000035314	Gdpd5	-Inf	0.038202
ENSMUSG00000020642	Rnf144a	-4.86493	0.038353
ENSMUSG00000034112	Atp2c2	-Inf	0.038403
ENSMUSG00000034310	Tmem132d	-3.49617	0.038565
ENSMUSG00000026039	Sgol2a	-Inf	0.038643
ENSMUSG00000020686	Gas2l2	-Inf	0.038655

ENSMUSG00000106757	Gm43482	-8.34306	0.038735
ENSMUSG00000005410	Mcm5	-Inf	0.03883
ENSMUSG00000049608	Gpr55	-Inf	0.038946
ENSMUSG00000001870	Ltbp1	-Inf	0.039155
ENSMUSG00000104687	Gm42899	-7.88735	0.03943
ENSMUSG00000021573	Tppp	-1.65531	0.039472
ENSMUSG00000022131	Gpr180	-5.43956	0.039562
ENSMUSG00000003437	Paf1	-2.98195	0.039738
ENSMUSG00000036432	Siah2	-Inf	0.039982
ENSMUSG00000033313	Fbxl8	-Inf	0.040066
ENSMUSG00000021120	Pigh	-Inf	0.040127
ENSMUSG00000105429	Gm43692	-Inf	0.040234
ENSMUSG00000039741	Bahcc1	-Inf	0.040382
ENSMUSG00000046312	AI464131	-Inf	0.04062
ENSMUSG00000037428	Vgf	-2.40664	0.04065
ENSMUSG00000052373	Mpp3	-2.09841	0.040669
ENSMUSG00000000489	Pdgfb	-4.90829	0.040958
ENSMUSG00000105936	Gm43544	-Inf	0.041122
ENSMUSG00000107045	Gm43636	-Inf	0.041806
ENSMUSG00000040710	St8sia4	-Inf	0.041855
ENSMUSG00000102919	Gm37726	-Inf	0.042251
ENSMUSG00000018820	Zfyve27	-2.38039	0.042628
ENSMUSG00000101599	Gm20342	-Inf	0.042706
ENSMUSG00000074766	Ism1	-Inf	0.043139
ENSMUSG00000074934	Grem1	-Inf	0.043144
ENSMUSG00000054855	Rnd1	-2.18594	0.043151
ENSMUSG00000021180	Rps6ka5	-Inf	0.043204
ENSMUSG00000067276	Capn6	-Inf	0.043241
ENSMUSG00000070047	Fat1	-Inf	0.043315
ENSMUSG00000102776	Gm38162	-8.72666	0.043371
ENSMUSG00000059991	Nptx2	-2.29347	0.043457
ENSMUSG00000056724	Nbeal2	-Inf	0.043469

ENSMUSG00000103770	Pcdha9	-Inf	0.043482
ENSMUSG00000020273	Papolg	-Inf	0.043548
ENSMUSG00000046191	Pcdhb20	-5.49458	0.044128
ENSMUSG00000028804	Csmd2	-1.88516	0.044138
ENSMUSG00000042156	Dzip1	-3.78533	0.044239
ENSMUSG00000043366	Olfr78	-Inf	0.04426
ENSMUSG00000052504	Epha3	-Inf	0.044267
ENSMUSG00000104138	Gm36949	-6.62198	0.044282
ENSMUSG00000049511	Htr1b	-Inf	0.044429
ENSMUSG00000031986	Sprtn	-Inf	0.04445
ENSMUSG00000058447	Gm26920	-7.15876	0.04447
ENSMUSG00000042292	Mkl1	-1.96727	0.044512
ENSMUSG00000049939	Lrrc4	-3.64862	0.044648
ENSMUSG00000041720	Pi4ka	-1.68469	0.044863
ENSMUSG00000063626	Unc5d	-2.77302	0.044883
ENSMUSG00000028780	Sema3c	-Inf	0.045068
ENSMUSG00000018339	Gpx3	-Inf	0.045456
ENSMUSG00000061578	Ksr2	-3.12939	0.04566
ENSMUSG00000107729	RP23-477A19.5	-Inf	0.045692
ENSMUSG00000027546	Atp9a	-2.21039	0.045708
ENSMUSG00000020672	Sntg2	-Inf	0.045712
ENSMUSG00000087178	A230056P14Rik	-Inf	0.045799
ENSMUSG00000053297	AI854703	-Inf	0.0459
ENSMUSG00000003665	Has1	-Inf	0.046041
ENSMUSG00000062794	Zfp599	-Inf	0.046041
ENSMUSG00000071573	Rnls	-Inf	0.046071
ENSMUSG00000043456	Zfp536	-4.34897	0.04658
ENSMUSG00000056458	Mok	-5.37798	0.046816
ENSMUSG00000052273	Dnah3	-Inf	0.04721
ENSMUSG00000069727	Gm5595	-Inf	0.047431
ENSMUSG00000096926	Gm26823	-Inf	0.047432
ENSMUSG00000025821	Zfp282	-Inf	0.047564

ENSMUSG00000028758	Kif17	-2.66794	0.048066
ENSMUSG00000043065	Spice1	-4.99372	0.048147
ENSMUSG00000063785	Utp14a	-4.4175	0.048355
ENSMUSG00000104737	Gm42937	-Inf	0.048364
ENSMUSG00000089957	A830011K09Rik	-Inf	0.048478
ENSMUSG00000027601	Mtfr1	-5.43476	0.048502
ENSMUSG00000097699	5430400D12Rik	-Inf	0.048772
ENSMUSG00000032806	Slc10a3	-Inf	0.048884
ENSMUSG00000073380	Arrdc5	-Inf	0.04897
ENSMUSG00000024413	Npc1	-2.50603	0.049223
ENSMUSG00000038600	Atp6v0a4	-Inf	0.049265
ENSMUSG00000056752	Dnah9	-Inf	0.049422
ENSMUSG00000040794	C1qtnf4	-Inf	0.049664
ENSMUSG00000031862	Atp13a1	-2.31286	0.049708

Supplementary Table 6. The list of genes (33) that are upregulated in Glu$^{S1L2/3}$ neurons of both CCI offspring and CCI maternal mice relative to those of sham controls (fold change ≥ 2, $P < 0.05$).

Ensembl Gene ID	Gene Symbol	Log2 Fold Change CCI offspring over sham	P value	Fold Change CCI over sham	P value
ENSMUSG00000075528	Aarsd1	6.6623	0.0000	6.024	0.0077
ENSMUSG00000022817	Itgb5	7.4764	0.0010	6.778	0.0470
ENSMUSG00000054675	Tmem119	3.9018	0.0010	4.256	0.0021
ENSMUSG00000041515	Irf8	+Inf	0.0012	+Inf	0.0187
ENSMUSG00000026841	Fibcd1	+Inf	0.0014	+Inf	0.0346
ENSMUSG00000036880	Acaa2	+Inf	0.0014	+Inf	0.0499
ENSMUSG00000024621	Csf1r	2.7652	0.0040	3.611	0.0069
ENSMUSG00000018930	Ccl4	2.8124	0.0052	2.919	0.0056
ENSMUSG00000032425	Zfp949	3.6700	0.0055	3.561	0.0459
ENSMUSG00000018008	Cyth4	+Inf	0.0055	+Inf	0.0458
ENSMUSG00000021238	Aldh6a1	4.7638	0.0059	5.287	0.0036
ENSMUSG00000046447	Camk2n1	1.7966	0.0075	2.200	0.0123
ENSMUSG00000034652	Cd300a	7.8475	0.0078	8.096	0.0135
ENSMUSG00000074622	Mafb	5.7386	0.0127	6.707	0.0016
ENSMUSG00000021665	Hexb	2.0263	0.0146	2.741	0.0083
ENSMUSG00000013523	Bcas1	+Inf	0.0154	+Inf	0.0007
ENSMUSG00000018593	Sparc	1.8281	0.0167	2.849	0.0086
ENSMUSG00000036887	C1qa	1.6703	0.0179	2.399	0.0180
ENSMUSG00000091387	Gcnt4	+Inf	0.0195	+Inf	0.0339
ENSMUSG00000031907	Zfp90	+Inf	0.0219	+Inf	0.0386
ENSMUSG00000036905	C1qb	1.5582	0.0232	2.192	0.0243
ENSMUSG00000001768	Rin2	6.1465	0.0244	8.712	0.0007
ENSMUSG00000037405	Icam1	4.7409	0.0246	5.337	0.0173
ENSMUSG00000060636	Rpl35a	6.2968	0.0253	7.697	0.0034
ENSMUSG00000022500	Litaf	4.2388	0.0281	5.342	0.0058
ENSMUSG00000032667	Pon2	3.6713	0.0310	4.142	0.0240
ENSMUSG00000029119	Man2b2	+Inf	0.0317	+Inf	0.0191
ENSMUSG00000031197	Vbp1	2.0402	0.0332	2.220	0.0475

ENSMUSG00000021532	Fastkd3	+Inf	0.0336	+Inf	0.0188
ENSMUSG00000044231	Nhlrc1	+Inf	0.0365	+Inf	0.0056
ENSMUSG00000002147	Stat6	+Inf	0.0399	+Inf	0.0223
ENSMUSG00000004837	Grap	6.1010	0.0437	7.349	0.0150
ENSMUSG00000024507	Hsd17b4	3.0556	0.0473	3.469	0.0417

Supplementary Table 7. The list of genes (69) that are downregulated in $\text{Glu}^{S1L2/3}$ neurons of both CCI offspring and CCI maternal mice relative to those of sham controls (fold change ≤ 0.5, $P < 0.05$).

Ensembl Gene ID	Gene Symbol	Log2 Fold Change CCI offspring over sham	P value	Log2 Fold change CCI over sham)	P value
ENSMUSG00000026224	4933407L21Rik	-9.6013	0.0000	-Inf	0.0002
ENSMUSG00000027400	Pdyn	-Inf	0.0003	-7.791	0.0058
ENSMUSG00000042216	Sgsm1	-3.2431	0.0003	-3.021	0.0066
ENSMUSG00000103472	Pcdhga7	-8.1558	0.0004	-Inf	0.0007
ENSMUSG00000038260	Trpm4	-Inf	0.0011	-Inf	0.0036
ENSMUSG00000046191	Pcdhb20	-Inf	0.0011	-5.495	0.0441
ENSMUSG00000027601	Mtfr1	-Inf	0.0018	-5.435	0.0485
ENSMUSG00000043456	Zfp536	-6.2548	0.0026	-4.349	0.0466
ENSMUSG00000025395	Prim1	-Inf	0.0037	-Inf	0.0090
ENSMUSG00000036834	Plch1	-5.1482	0.0062	-5.682	0.0222
ENSMUSG00000026404	Ddx59	-Inf	0.0068	-Inf	0.0153
ENSMUSG00000047415	Gpr68	-Inf	0.0071	-Inf	0.0143
ENSMUSG00000028661	Epha8	-Inf	0.0081	-Inf	0.0161
ENSMUSG00000039007	Cpq	-Inf	0.0081	-Inf	0.0174
ENSMUSG00000038214	Bend3	-Inf	0.0082	-Inf	0.0176
ENSMUSG00000035486	Plk5	-2.2501	0.0089	-2.729	0.0245
ENSMUSG00000033083	Tbc1d4	-6.4808	0.0098	-Inf	0.0021
ENSMUSG00000026198	Abcb6	-3.8647	0.0123	-4.635	0.0257
ENSMUSG00000085623	Gm16041	-Inf	0.0124	-Inf	0.0250
ENSMUSG00000105776	Gm43292	-6.9355	0.0134	-Inf	0.0088
ENSMUSG00000022894	Adamts5	-Inf	0.0137	-Inf	0.0235
ENSMUSG00000029648	Flt1	-Inf	0.0141	-Inf	0.0230
ENSMUSG00000039021	Ttc16	-Inf	0.0145	-Inf	0.0235
ENSMUSG00000024642	Tle4	-4.4459	0.0145	-6.633	0.0027
ENSMUSG00000031239	Itm2a	-Inf	0.0156	-Inf	0.0281
ENSMUSG00000029217	Tec	-Inf	0.0160	-Inf	0.0254
ENSMUSG00000103138	Gm2238	-8.7692	0.0162	-Inf	0.0193
ENSMUSG00000108353	RP24-144C5.1	-Inf	0.0163	-Inf	0.0291

ENSMUSG00000103348	Gm37053	-Inf	0.0164	-Inf	0.0302
ENSMUSG00000027164	Traf6	-3.5579	0.0172	-3.847	0.0232
ENSMUSG00000078815	Cacng6	-Inf	0.0173	-Inf	0.0263
ENSMUSG00000030823	9130019O22Rik	-7.2128	0.0180	-Inf	0.0097
ENSMUSG00000052221	Ppp1r36	-Inf	0.0200	-Inf	0.0340
ENSMUSG00000033740	St18	-Inf	0.0204	-Inf	0.0357
ENSMUSG00000016200	Syt14	-3.5213	0.0214	-7.565	0.0007
ENSMUSG00000038259	Gdf5	-5.4395	0.0214	-Inf	0.0030
ENSMUSG00000072720	Myo18b	-Inf	0.0217	-Inf	0.0333
ENSMUSG00000079553	Kifc1	-Inf	0.0217	-Inf	0.0329
ENSMUSG00000014782	Plekhg4	-Inf	0.0218	-Inf	0.0380
ENSMUSG00000034112	Atp2c2	-Inf	0.0222	-Inf	0.0384
ENSMUSG00000026039	Sgol2a	-Inf	0.0223	-Inf	0.0386
ENSMUSG00000049796	Crh	-Inf	0.0233	-Inf	0.0382
ENSMUSG00000033313	Fbxl8	-Inf	0.0238	-Inf	0.0401
ENSMUSG00000005410	Mcm5	-Inf	0.0239	-Inf	0.0388
ENSMUSG00000049608	Gpr55	-Inf	0.0241	-Inf	0.0389
ENSMUSG00000105936	Gm43544	-Inf	0.0243	-Inf	0.0411
ENSMUSG00000048537	Phldb1	-3.3931	0.0243	-Inf	0.0002
ENSMUSG00000107045	Gm43636	-Inf	0.0250	-Inf	0.0418
ENSMUSG00000016918	Sulf1	-Inf	0.0260	-Inf	0.0367
ENSMUSG00000034898	Filip1	-4.8039	0.0264	-Inf	0.0033
ENSMUSG00000103770	Pcdha9	-Inf	0.0278	-Inf	0.0435
ENSMUSG00000074867	Zfp808	-8.3597	0.0303	-Inf	0.0331
ENSMUSG00000102919	Gm37726	-Inf	0.0312	-Inf	0.0423
ENSMUSG00000061979	Wbscr16	-5.5264	0.0313	-Inf	0.0069
ENSMUSG00000022096	Hr	-5.1416	0.0333	-Inf	0.0033
ENSMUSG00000102854	C130023A14Rik	-6.0177	0.0339	-Inf	0.0109
ENSMUSG00000052273	Dnah3	-Inf	0.0352	-Inf	0.0472
ENSMUSG00000073380	Arrdc5	-Inf	0.0366	-Inf	0.0490
ENSMUSG00000058153	Sez6l	-1.4403	0.0403	-2.583	0.0072
ENSMUSG00000075415	Fnbp1	-1.6991	0.0433	-2.917	0.0108
ENSMUSG00000039116	Adgrg6	-6.6664	0.0445	-Inf	0.0238
ENSMUSG00000105083	Gm42699	-6.7940	0.0447	-Inf	0.0351

ENSMUSG00000018634	Crhr1	-3.2451	0.0459	-4.632	0.0197
ENSMUSG00000044072	Eml6	-2.5166	0.0460	-3.928	0.0176
ENSMUSG00000108522	RP23-385F8.2	-4.9597	0.0460	-Inf	0.0062
ENSMUSG00000046185	Zfp84	-4.6098	0.0478	-Inf	0.0031
ENSMUSG00000032396	Dis3l	-3.5290	0.0480	-6.636	0.0161
ENSMUSG00000044254	Pcsk9	-5.7847	0.0486	-Inf	0.0251
ENSMUSG00000075014	Gm10800	-2.2927	0.0495	-4.359	0.0005

97

Supplementary Table 8. qPCR primers used in this paper.

Primers used for mRNA qRT-PCR

Gene Symbol	Ensembl Gene ID	Forward primers	Reverse primers
Ccl4	ENSMUSG00000018930	CCACTTCCTGCTGTTTCTCT	TCTGTCTGCCTCTTTTGGTC
Epha4	ENSMUSG00000026235	TTGGGCAGATCGTCAAC	AGTCGCCCACTGATACTACA
Map2k2	ENSMUSG00000035027	AGCTGCCCAGTGGTGTGTTC	GCTTGATGAAGGCGTGGTTC
Trpc6	ENSMUSG00000031997	ATACAAAGGTCTGGCAAGTC	CAATGTTGGCAAGCACT
Camk2n1	ENSMUSG00000046447	ACGGCGACGAGAAGCTGAG	GCCCCGAAGAAGTTGTTGGT
Nfkbia	ENSMUSG00000021025	GACCTGGTTTCGCTCTTGT	CTGCTGTATCCGGGTACTTG
Pdpk1	ENSMUSG00000024122	CCCTTGGCACCAGTTTGT	AATGTGGCCCTTCTGTGAGT
Pik3r3	ENSMUSG00000028698	AGAGTGCGGACTGGTGGTC	CTCAGAAACTGTGGCATCCC
Birc2	ENSMUSG00000057367	CCTGTGCTCTAGCCCTCTTA	TCTGGCCTCTTCTGTACTCA
Adarb1	ENSMUSG00000020262	AACGGCTGCTCACCATGTC	GTACAGGCTGCCCAAGATGAT
C1qb	ENSMUSG00000036905	CACCTATCATGCCAGCTCC	ACCTCCTCTTGCTCTAGCTTCA
Epha8	ENSMUSG00000028661	ACCTGATCTCCAGCGTAAATG	CACTGCGTTGTAGGTGATGTC
Lrrn1	ENSMUSG00000034648	TTGTAATGACCTCCGCCTAA	CTTGGCGATGTTATTGCTCT
Pcdhb9	ENSMUSG00000051242	ATGAAGATGAGGACGTGCTTAC	CTGGCCCTTCTGCACAGT
Unc13a	ENSMUSG00000034799	GCGCCCAAGTATAACGAGA	GCGCAAAGCAGTAGTCCTTC
Acaa2	ENSMUSG00000036880	TGTTCAAGAAAGACGGGACAGT	CACGAAGTAGCCCACGACTC
Mafb	ENSMUSG00000074622	TGGAGGACCGCTTCTCTGAT	AATGAGCTGCGTCTTCTCGTT
Mtfr1	ENSMUSG00000027601	GAATGGAGTCCCGCACAC	TTCTGCCCTGAGTCTTATTGAG
Cpq	ENSMUSG00000039007	TGCCTCCCAGTATTATGAGCTA	CAGTCCAGTGGGTAAGAAGGTT
Flt1	ENSMUSG00000029648	CGGAAGACAGAAGTTCTCGTTA	GTCTAAGGTCGTAGAGCCACTG
Itm2a	ENSMUSG00000031239	ACCTGGTTGCTGTGGAAGAAAT	GCGTCTAAGGCGGAAGGAT
Tec	ENSMUSG00000029217	CGCCAAGTTCCCTGTGAAGT	TTCTTCTCAAAGGGCATCCTG
St18	ENSMUSG00000033740	GGGCCTATCAATGAGCAGAAT	CCGCCTGCTTGATGCTT

Primers used for ChIP-qPCR

Gene Symbol	Ensembl Gene ID	Forward primers	Reverse primers
Epha8	ENSMUSG00000028661	TATAATGCCCGGAAAGAAAGA	TCCTGATTGGCACAACCTT
Flt1	ENSMUSG00000029648	ACTCCGGGGGAGTGGATTA	AGCATGTCTCAATGCTCCC
Itm2a	ENSMUSG00000031239	TCATAATGAATCATATTAGGGTTG	TTTTTAGAGAGGAGGAAATTGTAT
Cpq	ENSMUSG00000039007	CAGGTGCAGAAGATTCAATAGAG	ACCTGGATGTTTTGAGTTAGGAT

Tec	ENSMUSG00000029217	GCAAGCCCATTTCTAAGCATT	ATATCCCTGCCCTGGCTG
St18	ENSMUSG00000033740	TTATTCGGCTTACACTTCCAT	GCTCCTGCTTTCTGACCTG
Mtfr1	ENSMUSG00000027601	GGACAAGTGGGACTGGATTTAT	GTATGAGGCTTAAGGAGTGGTTT
C1qb	ENSMUSG00000036905	GGCCTTCTTCCCTAGACTAT	GGTCCAGAGGCATGAGTT
Mafb	ENSMUSG00000074622	AGACTGCCAGGAGTGTGAGAC	GGTGACTTAGCCTCGTAGCTTT
Acaa2	ENSMUSG00000036880	TTCTGAGACAGTTTAGATTCCC	AATCAATCTTAACAAAAAGCCA

Supplementary Table 9. Statistical analyses.

Figure	Conditions	n per group	Analysis	F/t value	P value	Bonferroni post-hoc test
**Figure 1**						
**B**	sham vs CCI	5,12	Two-way RM ANOVA	F(1,15) = 68.783	P < 0.001	Day 0: P > 0.999<br>Day 3: P > 0.999<br>Day 5: P = 0.447<br>Day 7: P < 0.001<br>Day 14: P < 0.001<br>Day 21: P < 0.001<br>Day 28: P < 0.001
**C**	Mechanical: sham vs CCI (female)	41,50	Two-way ANOVA	F(1, 169) = 38.1	P < 0.001	P < 0.001
	Mechanical: sham vs CCI (male)	38,44				P = 0.124
	Thermal: sham vs CCI (female)	41,50	Two-way ANOVA	F(1, 169) = 42.89	P < 0.001	P < 0.001
	Thermal: sham vs CCI (male)	38,44				P < 0.001
**D**	Mechanical: sham vs CCI (female)	28,24	Two-way ANOVA	F(1, 94) = 12.14	P < 0.001	P < 0.001
	Mechanical: sham vs CCI (male)	23,23				P = 0.661
	Thermal: sham vs CCI (female)	28,24	Two-way ANOVA	F(1, 94) = 3.092	P = 0.082	P = 0.009
	Thermal: sham vs CCI (male)	23,23				P = 0.999
**E**	Mechanical: sham vs CCI (female)	21,28	Two-way ANOVA	F(1, 88) = 8.58	P = 0.004	P = 0.013
	Mechanical: sham vs CCI (male)	21,22				P = 0.340
	Thermal: sham vs CCI (female)	21,28	Two-way ANOVA	F(1, 88) = 11.59	P = 0.001	P = 0.003
	Mechanical: sham vs CCI (male)	21,22				P = 0.246
**F**	Mechanical: sham vs CCI	20,24	Unpaired two-tailed t test	t(42) = 5.642	P < 0.001	
	Thermal: sham vs CCI	20,24	Unpaired two-tailed t test	t(42) = 3.832	P < 0.001	
**G**	Mechanical: sham vs CCI	22,21	Unpaired two-tailed t test	t(41) = 4.669	P < 0.001	
	Thermal: sham vs CCI	22,21	Unpaired two-tailed t test	t(41) = 3.216	P = 0.003	
**Figure 2**						
**B**	sham offspring vs CCI offspring	22,26	Two-way RM ANOVA	F(1,46) = 10.656	P = 0.002	
**E**	sham offspring vs CCI offspring	14,18	Unpaired two-tailed t test	t(30) = 0.528	P = 0.601	
**F**	sham offspring vs CCI offspring	14,18	Two-way RM ANOVA	F(1,30) = 30.06	P < 0.001	

**I**	mCherry vs eNpHR	6,6	Two-way RM ANOVA	F(1,10) = 19.834	P = 0.001	BL: P > 0.999<br>Pre-light: P > 0.999<br>Light: P < 0.001
**L**	mCherry vs ChR2	7,7	Two-way RM ANOVA	F(1,12) = 55.474	P < 0.001	BL: P > 0.999<br>Pre-light: P = 0.781<br>Light: P < 0.001
**Figure 3**						
**B**	sham offspring vs CCI offspring	21,22	Unpaired two-tailed t test	t(41) = 6.667	P < 0.001	
**F**	AAV-control vs AAV-RNAi	8,7	Unpaired two-tailed t test	t(13) = 5.917	P < 0.001	
**G**	AAV-control vs AAV-RNAi	6,6	Two-way RM ANOVA	F(1,10) = 25.756	P < 0.001	D0: P > 0.999<br>D21: P < 0.001
**H**	AAV-control vs AAV-RNAi	7,11	Two-way RM ANOVA	F(1,16) = 32.555	P < 0.001	
**Figure 4**						
**D**	AAV-control vs AAV-MeCP2	6,6	Unpaired two-tailed t test	t(10) = 2.986	P = 0.014	
**E**	AAV-control vs AAV-MeCP2	6,6	Two-way RM ANOVA	F(1,10) = 31.532	P < 0.001	D0: P = 0.337<br>D21: P < 0.001
**F**	AAV-control vs AAV-MeCP2	11,15	Two-way RM ANOVA	F(1,24) = 28.942	P < 0.001	
**Figure 5**						
**B**	AAV-control vs AAV-RNAi	5,6	Two-way RM ANOVA	F(1,9) = 20.732	P = 0.001	D0: P > 0.999<br>D21: P < 0.001
**D**	AAV-control offspring vs AAV-RNAi offspring	8,12	Unpaired two-tailed t test	t(18) = 3.830	P = 0.001	
**E**	Mechanical: AAV-control offspring vs AAV-RNAi offspring	13,20	Unpaired two-tailed t test	t(31) = 2.779	P = 0.009	
	Thermal: AAV-control offspring vs AAV-RNAi offspring	13,20	Unpaired two-tailed t test	t(31) = 2.310	P = 0.028	
**F**	AAV-control offspring vs AAV-RNAi offspring	14,20	Two-way RM ANOVA	F(1,32) = 7.748	P = 0.009	
**H**	AAV-control vs AAV-MeCP2	8,9	Two-way RM ANOVA	F(1,15) = 19.494	P < 0.001	D0: P = 0.586<br>D21: P < 0.001
**J**	AAV-control offspring vs AAV-MeCP2 offspring	10,8	Unpaired two-tailed t test	t(16) = 2.717	P = 0.015	
**K**	AAV-control offspring vs AAV-MeCP2 offspring	17,21	Unpaired two-tailed t test	t(36) = 4.873	P < 0.001	

**I**	AAV-control offspring vs AAV-MeCP2 offspring	24,34	Two-way RM ANOVA	F(1,56) = 23.920	P < 0.001	
**M**	AAV-control vs AAV-RNAi	4,5	Two-way RM ANOVA	F(1,7) = 6.018	P = 0.044	D0: P = 0.512<br>D21: P = 0.0008
**Figure 6**						
**E**	correlation between RNA-seq log2(fold change) and qPCR log2(fold change)	5,5	Spearman's rank correlation test		r = 0.913<br>P < 0.001	
**H**	sham vs CCI (Epha8)	4,4	Paired two-tailed t test	t(3) = 1.916	P = 0.151	
	sham vs CCI (Flt1)	4,4	Paired two-tailed t test	t(3) = 12.351	P = 0.001	
	sham vs CCI (Itm2a)	4,4	Paired two-tailed t test	t(3) = 6.598	P = 0.007	
	sham vs CCI (Cpq)	4,4	Paired two-tailed t test	t(3) = 7.320	P = 0.005	
	sham vs CCI (St18)	4,4	Paired two-tailed t test	t(3) = 1.275	P = 0.292	
	sham vs CCI (Tec)	4,4	Paired two-tailed t test	t(3) = 0.272	P = 0.804	
	sham vs CCI (Mtfr1)	4,4	Paired two-tailed t test	t(3) = 6.940	P = 0.006	
**I**	sham vs CCI (C1qb)	4,4	Paired two-tailed t test	t(3) = 3.369	P = 0.043	
	sham vs CCI (Mafb)	4,4	Paired two-tailed t test	t(3) = 0.275	P = 0.801	
	sham vs CCI (Acaa2)	4,4	Paired two-tailed t test	t(3) = 3.198	P = 0.049	
**K**	sham vs CCI (Epha8)	6,6	Unpaired two-tailed t test	t(10) = 4.215	P = 0.002	
	sham vs CCI (Flt1)	4,6	Unpaired two-tailed t test	t(8) = 1.977	P = 0.084	
	sham vs CCI (Itm2a)	4,6	Unpaired two-tailed t test	t(8) = 4.283	P = 0.003	
	sham vs CCI (Cpq)	6,6	Unpaired two-tailed t test	t(10) = 3.156	P = 0.010	
	sham vs CCI (St18)	4,6	Unpaired two-tailed t test	t(8) = 4.359	P = 0.002	
	sham vs CCI (Tec)	4,6	Unpaired two-tailed t test	t(8) = 3.918	P = 0.004	
	sham vs CCI (Mtfr1)	6,6	Unpaired two-tailed t test	t(10) = 2.427	P = 0.036	
	sham vs CCI (C1qb)	6,6	Unpaired two-tailed t test	t(10) = 8.603	P < 0.001	
	sham vs CCI (Mafb)	6,6	Unpaired two-tailed t test	t(10) = 3.070	P = 0.012	
	sham vs CCI (Acaa2)	6,6	Unpaired two-tailed t test	t(10) = 2.061	P = 0.066	
**Supplementary Figure 1**						

**B**	sham vs CCI	6,6	Two-way RM ANOVA	F(1,10) = 134.901	P < 0.001	Day 0: P = 0.999<br>Day 7: P < 0.001<br>Day 14: P < 0.001<br>Day 21: P < 0.001<br>Day 28: P < 0.001
**C**	Mechanical: sham vs CCI	23,19	Unpaired two-tailed t test	t(40) = 1.262	P = 0.214	
	Thermal: sham vs CCI	23,19	Unpaired two-tailed t test	t(40) = 0.967	P = 0.339	
**D**	Mechanical: sham vs CCI	19,19	Unpaired two-tailed t test	t(36) = 1.435	P = 0.160	
	Thermal: sham vs CCI	19,19	Unpaired two-tailed t test	t(36) = 0.394	P = 0.696	
**Supplementary Figure 2**						
**A**	Time in center: sham vs CCI	13,19	Unpaired two-tailed t test	t(30) = 3.682	P < 0.001	
	Number of entries into center: sham vs CCI	13,19	Unpaired two-tailed t test	t(30) = 2.455	P = 0.020	
**B**	Time in open arms: sham vs CCI	13,19	Unpaired two-tailed t test	t(30) = 3.061	P = 0.005	
	Number of entries into open arms: sham vs CCI	13,19	Unpaired two-tailed t test	t(30) = 2.298	P = 0.029	
**Supplementary Figure 4**						
**B**	sham vs CCI	27,23	Two-way RM ANOVA	F(1,48) = 8.679	P = 0.005	
**D**	sham vs CCI	10,16	Unpaired two-tailed t test	t(24) = 4.383	P < 0.001	
**Supplementary Figure 5**						
**B**	sham vs CCI	30,21	Two-way RM ANOVA	F(1, 49) = 0.434	P = 0.513	
**D**	sham vs CCI	22,23	Unpaired two-tailed t test	t(43) = 1.048	P = 0.301	
**Supplementary Figure 6**						
**B**	sham vs CCI	25,25	Two-way RM ANOVA	F(1, 48) = 0.292	P = 0.591	
